# Bio-inspired 3D-printed earthen materials and structures

**DOI:** 10.1038/s41467-026-71885-z

**Published:** 2026-04-18

**Authors:** Samuel J. Armistead, Yierfan Maierdan, Olga B. Carcassi, Rebecca A. Mikofsky, Shiho Kawashima, Lola Ben-Alon, Wil V. Srubar III

**Affiliations:** 1 Department of Civil, Environmental, and Architectural Engineering, University of Colorado Boulder, Boulder, CO USA; 2 Department of Civil Engineering and Engineering Mechanics, Columbia University, New York, NY USA; 3 Graduate School of Architecture, Planning and Preservation, Columbia University, New York, NY USA; 4 Materials Science and Engineering Program, University of Colorado Boulder, Boulder, CO USA

**Keywords:** Civil engineering, Composites, Bioinspired materials

## Abstract

Nature provides a powerful blueprint for fabricating high-performance 3D-printed earthen materials and structures, such as termite mounds, wasp nests, and honeycomb worm reefs. However, mimicking the chemical and biological building blocks nature employs remains largely unexplored at scale. Here, we introduce a multiscale, bio-inspired approach that optimizes physicochemical interactions between biopolymers and earthen minerals at the microscale and systematically scales preferred interactions across spatial dimensions to fabricate macroscale, high-performance, 3D-printed earthen structures. By analyzing 90% of global subsoil minerals, we established a universally applicable multiscale optimization pathway, which converged on an alginate-based biopolymer-stabilizer that increases printing speeds by 33% and structural stability by 10° in architecturally relevant structures. This discovery accelerates the development of more efficient, resilient, and complex 3D-printed earthen structures, paving the way for sustainable, high-performance construction in the 21st century.

## Abstract (web summary)

Authors present a bio-inspired approach that, through multiscale optimization, converges on the use of alginate biopolymers to enhance 3D printed earthen structures, achieving a 33% increase in printing speed and a 10° improvement in structural stability over conventional methods.

## Introduction

Earthen architecture has provided durable and sustainable shelter for millennia^1^. Today, advanced fabrication technologies, such as 3D-printing, offer an opportunity to modernize this ancient practice for 21st-century construction^2^. In the context of the current climate crisis, which necessitates reducing our reliance on finite resources, earthen materials present a compelling solution^3^. Earthen materials are sustainable, non-toxic, and cost-effective, making them a promising option for promoting the adoption of low-carbon construction^4^. However, challenges remain, including inconsistent material properties and performance compared to traditional construction materials, hindering their widespread use^5^. Addressing these issues will require innovative approaches to engineering earthen materials with consistent, high-performance characteristics at scale^6^.

**Fig. 1:**
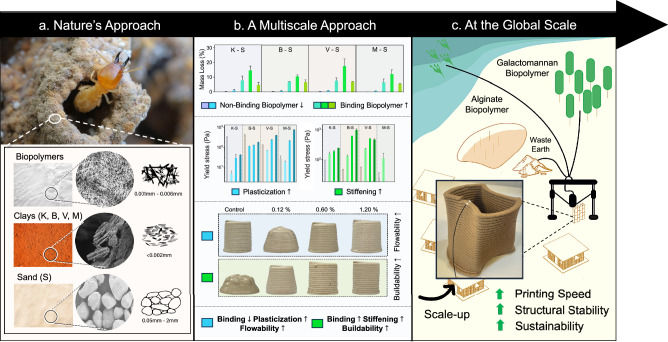
Bio-inspired 3D-printed earthen materials and structures. **a** Inspired by nature’s use of biopolymers and earth minerals to create robust structures, **b** a multiscale optimization approach has been developed which identifies the optimal interactions between biopolymers and earth minerals, mixture designs, and processing parameters. **c** This establishes the critical materials science foundation, a key step towards the production of high-performance 3D-printed earthen structures at the global scale.

Nature provides a blueprint for addressing these challenges. Having evolved mechanisms to sustainably manipulate earth minerals into high-performance structures, such as termite mounds, wasp nests and honeycomb worm reefs, living organisms produce a biological, biopolymer-based mortar to assemble mineral bricks in precise layers^7–10^. These biopolymers act as crucial mediators in material assembly (Fig. 1)^11^. Despite their promise, the intricate chemical and biological principles that nature utilizes to tune the properties of earthen materials remain largely unexplored. This knowledge gap arises from the absence of an experimental framework designed to elucidate and optimize the physicochemical interactions of biopolymers and earth minerals at the microscale and to systematically scale preferred interactions across spatial dimensions for the design and fabrication of high-performance, biopolymer-stabilized 3D-printed earthen materials and structures.

The promise of this bio-inspired strategy is reflected in the growing global use of biopolymer stabilizers to transform earthen materials into high-performance, sustainable construction materials^6^. In a recent study, Xu et al.^12^ demonstrated that mixtures of quaternized chitosan and alginate polysaccharides could stabilize diverse aggregates, including sand, slag, and crushed brick, attaining compressive and flexural strengths up to 17 MPa and 14 MPa, respectively. Kulshreshtha et al.^13^ created CoRncrete by mixing corn starch with sand, achieving strengths up to 26 MPa. Christ et al.^14^ discovered that the addition of gelatin biopolymers to sand generated compressive strengths of up to 60 MPa and revealed a correlation between adhesive strength and bulk compressive strength performance. While these studies report substantial strength gains over unstabilized earth (0.3–7 MPa), with values approaching or in some cases exceeding the minimum strength levels commonly associated with structural masonry (~14 MPa) and concrete (~20 MPa), they do not necessarily determine or report the underlying chemical and biological mechanisms that underpin the stabilization and property enhancements^15–17^.

Armistead et al.^18^ pioneered a new characterization protocol for earthen materials that elucidates the underlying chemical and biological interactions between minerals and biopolymers and links those interactions to bulk material properties. Using the protocol, the authors discovered that increased binding affinity between galactomannan biopolymers and iron oxide (Fe_2_O_3_) correlated directly with increases in compressive strength^19,20^. Mikofsky et al.^21^ employed the protocol to discover that biopolymer binding affinity was predominantly inversely correlated with compressive strength of bentonite and kaolinite clays. In Maierdan et al.^22^ the protocol was applied for the first time to study the influence of alginate on the 3D-printing properties of kaolinite, with rheological and small-scale extrusion experiments revealing that alginate-driven electrostatic repulsions improved the printing performance of kaolinite-based earthen materials. More recently, the protocol was further applied to locust bean gum-kaolinite systems, demonstrating that biopolymer bridging interactions can enhance underwater extrusion behavior in small-scale experiments^23^. While these advances are notable, the findings to date have been limited in (1) universality^18–23^ (i.e., applicability to multiple clays and biopolymer systems), (2) complexity^24^ (i.e., applicability to engineered or natural clay-sand systems), and (3) scale^2,25–27^ (i.e., applicability to macroscale 3D-printing).

**Fig. 2:**
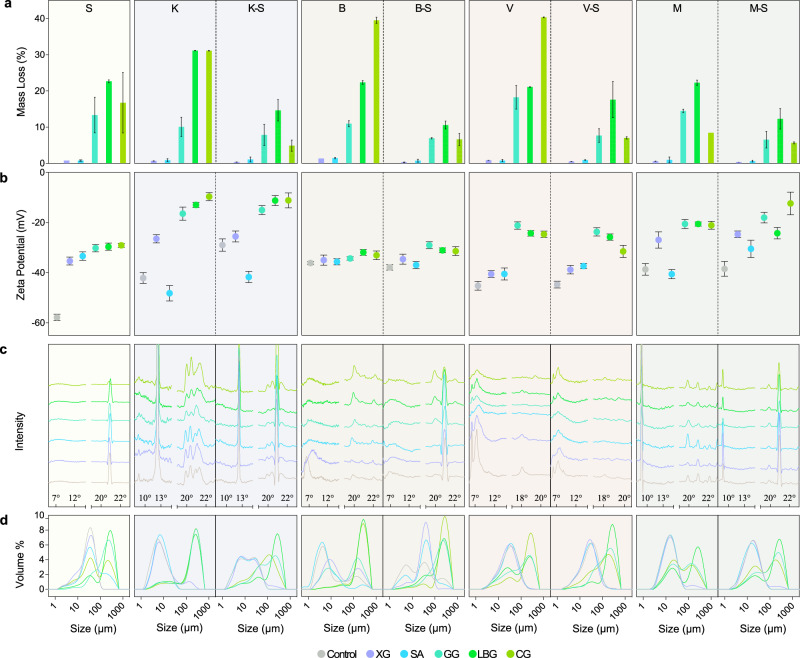
The binding characterization of biopolymer-mineral combinations for 3D-printed earthen materials. **a** Thermal gravimetric analysis between 200 and 400 °C (mean ± SD, *n* = 2) **b** Zeta potential (mean ± SD, *n* = 5) **c** X-Ray diffraction **d** Particle size distribution.

**Fig. 3:**
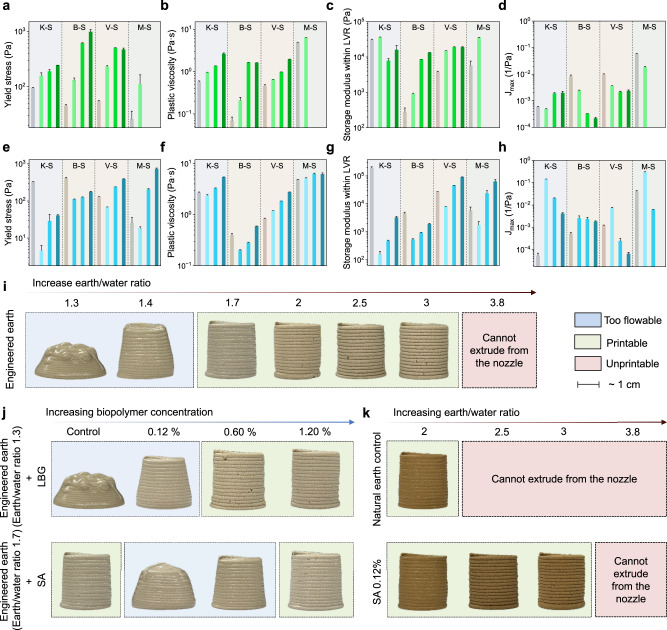
Rheological characterization of biopolymer-earth systems and small-scale 3D-printing of engineered and natural earthen materials. Influence of SA concentration on: **a** Yield stress **b** Plastic viscosity **c** Storage modulus **d** Creep compliance. Influence of LBG concentration on: **e** Yield stress **f** Plastic viscosity **g** Storage modulus **h** Creep compliance. Data (**a**–**h**) is presented as mean ± SD (*n* $\ge$ 2). **i** Printability of engineered earth with increasing solid content. **j** Printability of engineered earth with increasing biopolymer concentration. **k** Comparison of printability in natural earth with and without 0.12% SA with increasing earth-to-water content.

**Fig. 4:**
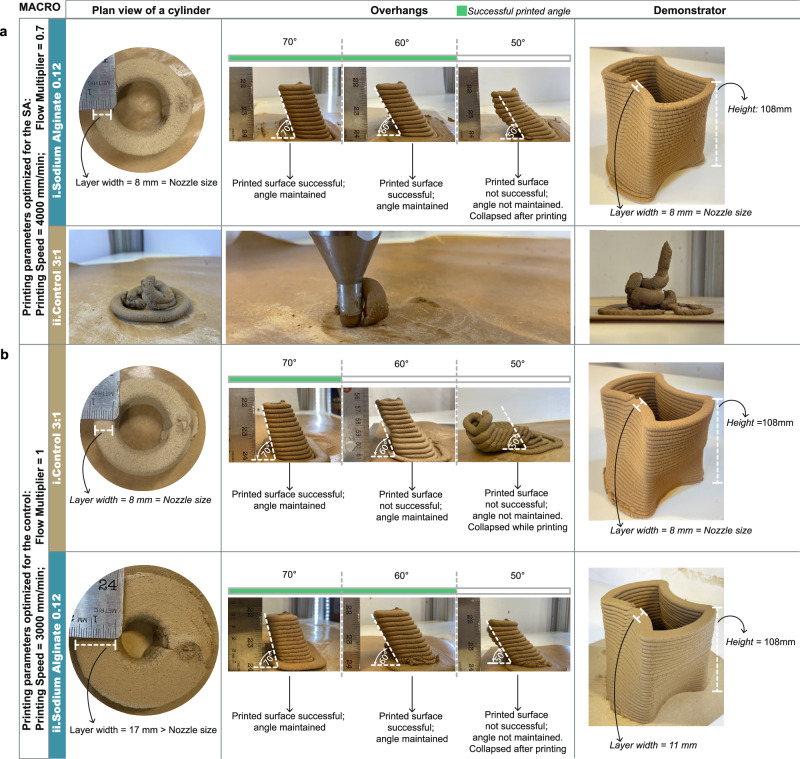
3D-printed earthen structures. **a** Printing parameters were optimized for SA 0.12 biopolymer-stabilized natural earth material (0.12% biopolymer-to-water, w/w) at a 3:1 earth-to-water ratio (w/w) to achieve an 8 mm layer wall and then tested on architectural relevant geometries. **b** Printing parameters were optimized for the control natural earth material at a 3:1 earth-to-water ratio (w/w) to achieve an 8 mm layer wall and then tested on architectural relevant geometries.

Here, we develop and deploy a multiscale framework to systematically investigate and optimize biopolymer stabilizers for 3D-printed earthen materials and structures. First, at the microscale, we establish a biopolymer-mineral binding characterization protocol to elucidate the fundamental physiochemical interactions between specific biopolymer functional groups and earth minerals (Fig. 2). This approach identified promising biopolymer-earth combinations for further exploration. Second, at the mesoscale, rheological analysis was employed to examine the fresh-state behavior, providing critical insights into material flowability, stiffness, and stability (Fig. 3a-h), thereby determining promising biopolymer-earth combinations and biopolymer-to-water ratios for further exploration. To verify these findings and to establish the optimal earth-to-water ratio, small-scale 3D-prints were conducted, focusing on printability (i.e., the ability to smoothly extrude from a nozzle and form stable, layered structures over time) (Fig. 3i-k). Finally, at the macroscale, the selected biopolymer stabilizers and optimized ratios were assessed for their effectiveness in enhancing printing performance, including processing parameters and print quality, of natural, heterogeneous earthen materials in 3D-printing (Fig. 4).

## Results and discussion

### Earth mineral selection

Subsoils (0.3–2 m), often generated as construction waste, can be repurposed for earthen material production. Globally, these subsoils typically consist of a non-clay fraction (50–90%)^28^ predominantly composed of quartz minerals, and a clay fraction (10–50%), which includes four primary minerals: kaolinite (28%), bentonite (15%), vermiculite (8%), and mica (28%)^29^. These layered phyllosilicates are defined by their tetrahedral silica sheets and octahedral alumina sheets, with kaolinite (K) exhibiting 1:1, and bentonite (B), vermiculite (V), and mica (M) exhibiting 2:1 tetrahedral:octahedral sheet ratio^30^. To assess the applicability of biopolymer stabilizers at a global scale, all four clay minerals were analyzed alongside sand (S), using a 2:1 sand-to-clay ratio to approximate an average system. The study therefore, explores 90% of subsoil minerology, providing critical sensitivity insights for regional and local studies throughout the globe. The composition and chemistry of the sand and clay materials are detailed in Supplementary Figs. 1, 2, 3b, discussed in Supplementary 1.1.1^31–37^, and visually represented in Supplementary Fig. 3a.

### Biopolymer selection

Nature synthesizes optimal biopolymer stabilizers in situ through biosynthetic, metabolic, and secretion-based processes tailored to specific mineralogy^38^. While nature intuitively selects biopolymer stabilizers through an inherent understanding of the underlying chemistry and biology, in this work, the outlined multiscale framework aims to systematically elucidate these phenomena. Therefore, polysaccharides were selected primarily for their diverse chemistries while also considering variability in production methods and potential for rapid scalability. Galactomannans (e.g., guar gum (GG), locust bean gum (LBG), and cassia gum (CG)) are non-ionic polysaccharides derived from legumes grown in arid, moderate-temperature regions, which are produced at approximately 1,500,000 tons annually^39^. Sodium alginate (SA), an anionic polysaccharide sourced from brown seaweed, is produced at 23,000 tons annually^40,41^. SA has previously shown promise in 3D-printed earthen materials^42^. Xanthan gum (XG), a highly branched anionic bacterial exopolysaccharide, is produced at 80,000 tons annually via bio-fermentation in modular, climate-independent micro-fermenters^43^. Biopolymer characterization and chemistry are outlined in Supplementary Fig. 5a, discussed in Supplementary 1.1.2^44–49^ and visually represented in Supplementary Fig. 5b, c.

### Microscale optimization

Non-ionic biopolymers (*i.e*., GG, LBG, CG) exhibit significant binding to clays, as evidenced by mass loss in TGA (Fig. 2a). ATR-FTIR analysis confirms the binding of non-ionic biopolymers through characteristic peaks at ~1650 cm^−1^ and ~3000 cm^−1^ with minimal shifts compared to biopolymer controls (Supplementary Fig. 6). These results indicate that hydrophobic forces, rather than electrostatic or covalent bonding, drive these interactions through the affinity between the hydrophobic mannose groups of galactomannans and the siloxane basal planes of clay minerals (Supplementary Fig. 7). Among non-ionic biopolymers, CG demonstrates superior affinity for clays (Fig. 2a) due to its higher proportion of mannose groups relative to LBG or GG (Supplementary Fig. 5c). However, when minerals containing highly charged surfaces, such as sand, are introduced, CG’s binding affinity is reduced (Fig. 2a). This decrease is likely due to CG’s highly hydrophobic mannose domains, which are repelled by highly charged surfaces (Supplementary Fig. 7). LBG, due to its moderate number of mannose groups displays consistent performance across clay-sand systems (Fig. 2a). These characteristics position LBG as a promising binding biopolymer candidate for further investigation. In addition, these findings highlight a critical trend: binding affinities in clay-sand systems align more closely with sand systems than with clay alone, underscoring the pivotal role of sand in understanding the properties and performance of biopolymer-stabilized earth. This finding directly challenges the conventional belief in civil engineering that sand serves only as an inert filler.

Anionic biopolymers (i.e., XG, SA) show negligible interaction with clays, indicated by non-significant mass losses in TGA (Fig. 2a). Although non-binding, SA disperses clay microstructures as revealed by XRD analysis (Fig. 2c). Additionally, zeta potential surface charge shifts occur: kaolinite exhibits a more negative charge, while vermiculite and mica exhibit a more positive charge (Fig. 2b). These effects result from the disruption of intermolecular interactions between clay structures, exposing negatively charged edge groups in kaolinite and positively charged edge groups in vermiculite and mica clays (Supplementary Fig. 7). In contrast, the highly branched structure of XG limits its ability to penetrate clay structures, restricting its capacity to induce significant microstructural changes (Fig. 2c). The addition of sand has minimal impact on the binding affinity or physicochemical environment across clay-sand systems, attributed to the lack of interaction of SA and XG in sand-only systems (Fig. 2a). This finding underscores the importance of biopolymer structure, alongside charge, positioning it as a crucial microscale design parameter for future studies. SA’s ability to influence both microstructural and surface charge characteristics highlights its potential to serve as a dispersive, non-binding biopolymer. These characteristics render SA a compelling candidate for further investigation.

Independent of binding, 2:1 clays exhibit distinct microstructural changes upon the addition of biopolymers (Fig. 2c). Low-layer-charge bentonite (-0.36, Supplementary Fig. 3b) undergoes significant exfoliation, characterized by a loss of interlayer and clay-clay tactoid structures (Supplementary Fig. 7b). Medium-layer-charge vermiculite (−0.78, Supplementary Fig. 3b) retains its interlayer structure but exhibits exfoliated tactoid structures (Supplementary Fig. 7c). In contrast, high-layer-charge mica (−0.87, Supplementary Fig. 3b) remains largely unaffected, as indicated by minimal shifts in XRD peak position and profile (Supplementary Fig. 7d). These findings have important implications. Upon biopolymer addition, bentonite exhibits significant changes in particle size: it increases with binding biopolymers and decreases with non-binding polymers, as exfoliated clay layers enhance the surface area available for biopolymer interaction (Fig. 2d). Mica, by contrast, shows fewer particle size changes but exhibits greater charge shifts (Fig. 2b). This behavior is attributed to its lower surface area, resulting from preserved interlayer and tactoid structures, which allow biopolymers to shield its highly charged basal planes more effectively. These findings are significant, revealing the previously unrecognized critical role of clay layer charge in governing microstructural and physicochemical interactions within biopolymer-stabilized earthen materials.

### Mesoscale optimization

LBG and SA were selected for their promising binding and non-binding characteristics, respectively, to evaluate their impact on flow behaviors and bulk printing properties. While the primary focus is on biopolymer-induced changes, the baseline properties of clay systems are discussed in Supplementary 1.2^50–52^.

In 2:1 clay-sand systems (bentonite-, vermiculite-, and mica-sand), the addition of LBG increased the yield stress and storage modulus within the linear viscoelastic region (LVR), and reduced creep compliance (Fig. 3a, c, d), indicating the formation of a stronger and stiffer micro-fabric. This improvement is attributed to LBG’s function as a binding polymer (Fig. 2a), which adsorbs onto the surfaces of clay and sand particles, facilitates particle bridging, and forms larger flocs (Fig. 2d) that reinforce the overall micro-fabric. On increasing LBG concentration, bentonite-sand systems show increased sensitivity when compared to the other clay-sand systems, due to its highly exfoliated structure (Fig. 2c), resulting in a high availability of surface sites for binding biopolymers to bridge (Supplementary Fig. 7b).

Although all clay-sand systems show enhanced micro-fabric strength with LBG (mica-sand systems with high LBG concentrations are presented in Supplementary Fig. 9 m-r), kaolinite-sand systems exhibit a distinct three-phase viscoelastic trend. The key chemical difference between kaolinite and 2:1 clays is the accessibility of aluminum surfaces, which influences its micro-fabric by forming extensive face-to-edge (F–E) interactions (Supplementary Fig. 3a). Therefore, initially at lower LBG concentrations (0.12%), adsorption on siloxane surfaces stiffens the micro-fabrics by introducing additional binding between particles, increasing storage modulus, and reducing creep compliance (Fig. 3c, d). At intermediate concentrations (0.6%), LBG adsorption on aluminum surfaces disrupts the stiff clay-clay F–E interactions, reducing the stiffness of the network, resulting in a significant drop in storage modulus and an increase in creep compliance (Fig. 3c, d). At high concentrations (1.2%), excess LBG causes a recovery in storage modulus and further reduced creep compliance (Fig. 3c, d). Notably, the trends observed here are consistent regardless of how the biopolymer concentrations are defined—whether based on clay content or water content (Supplementary Fig. 9a-h). These findings mark a significant advancement in our understanding of the effects of binding biopolymers on the rheology of earthen materials. Further, they highlight the importance of rheological analysis in understanding biopolymer-earth interactions, particularly in systems like kaolinite-sand, where clay-clay interactions can influence micro-fabric behavior at varying biopolymer concentration.

As illustrated in Fig. 3e-h, at a low concentration (0.12%), the addition of non-binding SA induces a pronounced dispersion effect across all clay-sand types. This effect is evidenced by a substantial reduction in yield stress (Fig. 3e) and storage modulus within the LVR (Fig. 3g), along with a marked increase in creep compliance (Fig. 3h). The effect is particularly significant in the kaolinite-sand system, attributed to alterations in kaolinite’s micro-fabric by additional exposed negatively charged edge groups in suspension (Supplementary Fig. 7a), as evidenced by a significant drop in zeta potential (Fig. 2b). The greater number of negative surfaces results in a significant increase in electrostatic repulsion forces, thereby accounting for the dispersive effects observed.

Within the 2:1 clay-sand systems, SA had the most pronounced impact on bentonite-sand’s rheology, stemming from the disruption of bentonite’s microstructure at the platelet level, as evidenced by the strong exfoliation (Fig. 2d). This dispersion effect resulted in decreased shear response, increased creep, and reduced storage modulus across all concentrations (Fig. 3g, h, Supplementary Fig. 8c). In contrast, SA had a more moderate effect on vermiculite- and mica-sand systems (Fig. 3e-h, Supplementary Fig. 8e, g), primarily affecting their particle interactions at the tactoid or assembly level (Fig. 2b-d). For all clay-sand systems at higher SA concentrations, polymer overlapping effects dominated, increasing yield stress, viscosity, and storage modulus, while decreasing creep (Fig. 3e-h). Overall, the effectiveness of SA in dispersing clay particles depended on the strength of the clay-clay particle interactions, with the disruption of stronger interactions leading to more significant rheological changes. By examining a range of clay-sand systems, this study reinforces micro-scale findings, identifying clay layer charge as the critical factor governing earth microstructures and the rheological sensitivity of both binding and non-binding biopolymer-stabilized systems.

Small-scale prints were conducted to validate the rheological findings and optimize material composition for macroscale printing. The process began with an engineered earth modeled after a natural system (Supplementary Fig. 10, Supplementary Table S3.3), serving as a transitional step towards a natural earth system. Since LBG and SA have contrasting effects on material properties, different solid contents were selected to emphasize their effect on printing status. As previously outlined, the addition of LBG consistently increased the yield stress and storage modulus of 2:1 clay systems, which dominate the composition of the engineered earth (Supplementary Fig. 10f). This stiffening effect resulted in improved buildability with increasing LBG concentrations (Fig. 3j). In SA mixtures, consistent with rheological observations, the buildability of the mixtures initially decreased due to SA’s dispersion effect but gradually improved as polymer overlapping effects became dominant (Fig. 3j). Importantly, buildability can also be increased by raising the solid content of the mixture (Fig. 3i). Therefore, SA’s ability to enhance flowability at the same earth-to-water ratio enables the printing of mixtures with higher solid content, which potentially reduces drying shrinkage and improves the strength of the printable mixtures^22^. From this perspective, SA at a concentration of 0.12% was taken to represent the optimal biopolymer-to-water ratio for further development.

To validate SA’s effects in natural systems, small-scale printing experiments were conducted using natural earth with 0.12% SA at varying earth content. Consistent with engineered earth, SA improved flowability and enabled printing at significantly higher earth contents (Fig. 3k). This formulation resulted in a 75% reduction in drying shrinkage (from 10.31% to 2.61%) and a 25% improvement in compressive strength compared to control prints (Supplementary Fig. 11). Thus, SA 0.12 % and an earth-to-water ratio of 3:1 was used as the starting point for the macroscale investigation (Fig. 4).

### Macroscale optimization

At the macroscale, the experimental approach systematically explores key 3D printing processing parameters, complemented by a complex geometric demonstrator (Supplementary Fig. 10k). Printing parameters were optimized to achieve consistent 8 mm width layer walls (Fig. 4). Using the multiscale optimized material parameters of 0.12% SA biopolymer-to-water and a 3:1 earth-to-water ratio, a printing speed of 4000 mm/min, a flow multiplier of 0.7, and air pressure of 4 bar were achieved (Fig. 4ai). The SA 0.12% mixture successfully printed overhangs at 70° and 60° angles, though the 50° angle collapsed during printing. In contrast, the control material could not be extruded and printed under these conditions (Fig. 4aii).

When testing the control material further, adjusted parameters of a printing speed of 3000 mm/min, air pressure of 4 bar, and a flow multiplier of 1 enabled the creation of 8 mm layer walls and a 70° overhang (Fig. 4bi). However, the material failed at 60° and 50°, with the latter collapsing immediately. Applying these printing parameters to the SA 0.12% mixture, the layer wall thickness was increased from 8 mm to 17 mm. With this increased material deposition, a maximum overhang of 60° was successfully achieved together with the demonstrator (Fig. 4bii). These results reinforce findings from the micro- and meso-scales, highlighting that improved flowability, due to SA’s dispersion effects, drives the enhanced performance of SA biopolymer-stabilized 3D-printed earthen materials.

Taken together, these findings are significant: the multiscale optimized SA biopolymer-stabilized earth demonstrates a 33% increase in 3D-printing speed and enhances structural stability by 10°, which, in turn, can increase construction speed, strength, durability, and the ability to print macroscale geometries which were previous considered unachievable. These improvements highlight the potential of the multiscale approach for advancing high-performance 3D-printed earthen materials and structures at the macroscale. The dispersion effect of SA biopolymer, independent of the minerology in controlled or heterogenous natural earth, constitutes a foundational advancement in facilitating the widespread adoption of 3D-printing technology for earthen construction.

### Future impact

By designing and implementing a multiscale optimization framework, the performance of 3D-printed earthen materials and structures has been significantly enhanced, characterized by faster production rates and improved structural stability. This framework has demonstrated its ability to generate foundational insights across multiple scales and disciplines, ultimately converging on an optimal alginate-based biopolymer stabilizer to improve the 3D-printing properties of diverse and heterogeneous natural earth materials. The framework’s ability to quantitatively assess and optimize for the universality, complexity, and scalability of biopolymer-stabilized earth represents a key step toward the production of high-performance, 3D-printed earthen structures at the global scale.

Future studies can extend this approach to a broader range of processing parameters (i.e., structural build-up, thixotropy) and material properties (i.e., density, mechanical performance, thermal conductivity, and moisture resistance) under relevant environmental exposure conditions (i.e., humidity, temperature, and UV radiation), which are critical for the practical implementation of 3D-printed biopolymer-stabilized earth as a structural and/or insulative construction material. Importantly, the framework can be applied to optimize compressive strength, providing a pathway toward realizing the high strengths previously observed in bio-stabilized earth (up to 60 MPa) within 3D-printed structures. In addition to 3D-printed structures, the framework can also be employed to optimize the design and engineering of other earth-based construction methods, including rammed earth walls and compressed earth blocks. By integrating these advancements, researchers, engineers, architects, and builders can develop sustainable solutions that revolutionize construction by using locally sourced earthen materials in place of other resource- and carbon-intensive alternatives.

Furthermore, the multiscale framework provides a foundation for integrating cutting-edge technologies such as synthetic biology (i.e., engineered bio-stabilizers), digital processing (i.e., in situ imaging and analysis), and artificial intelligence (i.e., predictive performance modeling). This integration will enable the real-time analysis and optimization of large volumes of waste earth with tailored biopolymer stabilizers, an essential step toward the building-scale implementation in insulation, non-load-bearing, and load-bearing wall assemblies. When further coupled with supply chain, technoeconomic, and environmental analyses, these innovations collectively will provide a foundation for the widespread adoption of bio-inspired, 3D-printed earthen materials and structures.

## Method

### Materials

Xanthan gum (CAS 11138-66-2), sodium alginate (CAS 9005-38-3), guar gum (CAS 9000-30-0), and locust bean gum (CAS 9000-40-2) were obtained from Sigma-Aldrich. Cassia gum (CAS 11078-30-1) was sourced from Premcem Gums Private Ltd. Natural kaolinite (CAS 1318-74-7), bentonite (CAS 1302-78-9), and vermiculite 2–3 mm (CAS 1318-00-9) were sourced from Sigma-Aldrich. Mica powder (CAS 12001-26-2) was obtained from MakingCosmetics Inc. ASTM (C778) graded Ottawa sand from Humboldt Mfg. Co. was obtained and used throughout. Silty Ottawa Sand F-75 from Krueger Potter was sourced and used specifically for the engineered earth prints. The natural earth used was a byproduct of excavation operations sourced from a granite quarry in Golden, Colorado (USA). In the materials characterization and microscale testing, ultra-pure deionized water from a Milli-Q Direct-8 Q UV-R water purification system was used. The mesoscale testing used distilled water, while the macroscale testing used water derived from New York City water system.

### Material characterization and preparation

#### Biopolymer characterization

A ThermoScientific Nicolet iS20 Fourier Transform Infrared Spectrometer (FTIR) with a Diamond Attenuated Total Reflection (ATR) accessory was used to characterize biopolymer powders (Supplementary Fig. 5a). All measurements were performed with 256 scans and a resolution of 4 cm^-1^.

#### Clay and sand characterization and preparation

Kaolinite (K), bentonite (B), vermiculite (V), and mica (M) clays were processed by baking at 100 ± 10 °C for at least 5 h to remove excess moisture. Then, the clays and sand were ground (one minute with a mortar and pestle for K, B, and M; 10 min at 450 rpm in a Retsch ball mill for V and S) and sieved with a Gilson sieve shaker to <45 μm (No. 325 sieve). The mineral composition of the sand and clays was confirmed using powder X-ray Diffraction (XRD), attenuated total reflection Fourier transform infrared spectroscopy (ATR-FTIR), Thermal gravimetric analysis (TGA), and X-Ray Fluorescence (XRF). Clay layer charge was determined using the structural formula method^53^. Particle size and morphology were characterized using scanning electron microscopy (SEM) and particle size analysis (PSA). The following section outlines the specific instruments and protocols used.

Powder XRD was performed from 5 to 75° at 0.0194° step, 2 s/step (0.15 s/step for mica), with a 0.1 (K, B) or 0.2 mm (V, M, and S) divergence slit on a Bruker D8 Advance A25 with Cu source and Lynxeye XE-T detector. Back-loading was used to achieve random orientation, and a 30°/min rotation was used to obtain a good sampling. Unground sand was characterized by using front-loaded XRD to accommodate its larger particle size to ensure that the chemical composition remained unchanged upon grinding. Diffractograms for each mineral are shown in Supplementary Fig. 1a. FTIR was performed with a ThermoScientific Nicolet iS20 FTIR using a diamond crystal ATR accessory, 256 scans, and a 4 nm^−1^ resolution. FTIR spectra agreed with the literature (Supplementary Fig. 1b)^30^. TGA was conducted from 50 °C to 900 °C using a TA Discovery TGA 5500 in an N₂ gas atmosphere, with a ramp rate of 10 °C/min (Supplementary Fig. 1c). The particle size distributions were measured using a Malvern Panalytical Mastersizer 3000 outfitted with a HydroMV accessory under constant stirring and sonication with ~5–15% laser obscuration, a 1.55 refractive index, a 0.01 absorptive index, and general purpose analysis with fine powder mode (Supplementary Fig. 1d). Scanning electron micrographs were taken using a Hitachi SU3500 VP Scanning Electron Microscope (SEM) operating with a 5.0 kV accelerating voltage and spot size of 30. Samples were coated with ~7–8 nm of platinum prior to imaging (Supplementary Fig. 2).

#### Natural earth

Upon receiving the natural earth, it was first poured into containers and completely submerged in water. A 425 μm sieve was used to remove large aggregates that could hinder the printing process. After wet-sifting, the earth was left to settle naturally at the bottom of the container. The supernatant was carefully removed, leaving behind the saturated, sifted earth. To accelerate drying, grid-like pockets were created in the earth, and fans were employed to enhance airflow across all layers. The processed earth was then stored in a cool, dry area for at least seven days. Before further use, the dried earth was manually pulverized and dry-sieved to a particle size of less than 425 μm. The prepared natural earth was characterized using FTIR, TGA, PSA, and SEM, as described in the clay and sand characterization section (Supplementary Fig. 10a–c, g–i). For PSA, an obscuration of ~19–23% was selected to achieve stable results.

Due to its heterogenous nature, a more detailed XRD analysis was performed to characterize the natural earth mineralogy. The clay portion was first separated for XRD testing by washing (adding solution, centrifuging for 5 min at 2000 rpm, then decanting the supernatant) in 1 M NaCl twice, followed by deionized water washes until the supernatant was cloudy^54^. XRD samples were prepared by first suspending the clay portion in a small amount of deionized water, pipetting onto a 25 mm glass square, and drying overnight. Multiple samples were taken to account for heterogeneity. To determine the clay minerals present in the natural earth, samples were tested untreated and then either treated in a chamber with ethylene glycol (EG) vapor at 60 °C overnight or heated to 300 °C for at least 2 h and then heated to 550 °C for at least 30 min in a muffle furnacet^55^. All samples were then measured from 2 to 68° at 0.0194° step, 0.6 s/step, and 0.1 mm divergence slit on a Bruker D8 Advance A25 with Cu source and Lynxeye XE-T detector. The peak shifts due to these treatments were used to inform the clay composition for the quantitative analysis (Supplementary Fig. 10d).

For quantitative XRD analysis, the prepared natural earth was back-loaded to achieve random orientation and measured from 2 to 75° at 0.0194°/step and 2 s/step with a 0.1 mm divergence slit without rotation due to the low starting angle. The low-angle portion of the scan (2-10°) was used to check for 001 peak positions but not used for the quantitative analysis due to the influence of machine settings on intensity at these angles. Quantitative XRD Rietveld refinement was performed from 10 to 65° using Profex-BGMN. The instrument configuration was adjusted to match the machine settings, and the refinement parameters were adjusted as necessary to reduce the χ^2^ fit factor to account for clay anisotropy. The refinement results are shown in Supplementary Fig. 10e,f.

### Microscale methods

While the protocol was adapted from the authors’ previous work, the inherent complexity of clay systems necessitated significant advancements in earthen material and pore solution characterization^18–20^.

#### Biopolymer solution preparation

Biopolymer powder (100 mg) was first added to a 40 °C water solution under constant stirring to encourage dispersion of solid particulates. The solution was kept at 40 °C with constant stirring for 10 min, and then sonicated for 10 min, to encourage dissolution. All solutions were then stored in the dark and used within 24 h to prevent degradation.

#### Binding experiment

Prepared clay (50 mg), sand (100 mg), and dry-mixed clay-sand (clay: 50 mg, sand: 100 mg) were added to 20 mL of the prepared biopolymer solution in centrifuge tubes. The mixtures were vortexed for 30 s, followed by 10 min of sonication to disperse the minerals within the solution. A Labnet Mini LabRoller Rotator was then used to agitate the suspension for 30 min to facilitate binding. The suspension was then centrifuged at 3000 x *g* for 10 min, and the supernatant was carefully removed. The resulting pellet was washed four times by adding 50 mL of water, vortexing, centrifuging at 3000 x *g* for 10 min, and then removing the supernatant. This process ensured the removal of non-bound or transiently bound biopolymers that could artificially elevate the quantity of bound biopolymer. The final sample was then dried under ambient conditions and stored in glass vials until further testing. The pH (Hanna GroLine Soil pH Tester) and electrical conductivity (Bante Instruments 540 Portable Conductivity Meter) were measured for the initial biopolymer solutions, after mineral dispersion, and following agitation at the 30-minute time point to assess changes in solution chemistry (Supplementary Fig. 4).

#### Binding characterization

The resulting samples were analyzed using TGA, FTIR, Zeta Potential analysis, XRD, and PSA. Thermal analysis was conducted from 50 °C to 900 °C using a TA Discovery TGA 5500 in an N₂ gas atmosphere, with a ramp rate of 10 °C/min. Mass loss between 200 °C and 400 °C was used to quantify the amount of bound biopolymers, providing a measure of the affinity between biopolymers and earth minerals. Bonding interactions between biopolymers and earth minerals were characterized using a ThermoScientific Nicolet iS20 FTIR with a diamond crystal ATR accessory, utilizing 64 scans at a resolution of 4 cm⁻¹. XRD analysis was performed to investigate biopolymer-induced changes in mineral microstructures. The analysis used powder loading on a zero-background silicon plate with a Rigaku SmartLab 9 kW Cu source, operating from 2° to 90° at 0.5° per step and 20 steps per minute, with 2.5° Soller slits and a 2 mm length-limiting slit. Zeta potential analysis was performed to assess changes in surface charge using a Malvern Panalytical Zetasizer ZS with a folded capillary cell. Samples were suspended in a 10 mM KNO₃ solution at 1 g/L, sonicated for 10 min, diluted tenfold, and re-sonicated to achieve a 0.1 g/L concentration. Particle size distribution analysis was conducted to quantify changes in particle size using a Malvern Panalytical Mastersizer 3000 with a HydroMV accessory, operating with constant stirring, ~0.1–6% laser obscuration, a refractive index of 1.55, and an absorptive index of 0.01. For both zeta potential and PSA analyses, the pH of the measurement solution was adjusted to replicate binding conditions, where necessary (B–pH 9, V–pH 8, Supplementary Fig. 4), using a 2 M NaOH solution. Together, these techniques provide a comprehensive overview of the effects of biopolymers at the molecular level.

### Mesoscale methods

#### Rheology mix proportions

Rheological investigations were conducted to evaluate the effects of different biopolymer types—binding LBG and non-binding SA—and varying biopolymer concentrations on the rheological performance of clay-sand systems. The clay-to-sand ratio was fixed at 1:2 across all experiments to maintain consistency.

As shown in Supplementary Table S3.1, to ensure that all groups remained within the measurement range of the rheometer, different earth-to-water (w/w) ratios were selected based on the specific characteristics of each biopolymer and clay type and preliminary testing. For instance, SA’s dispersion effect on clay required a higher earth-to-water ratio for the control groups, whereas LBG, which increases the stiffness of the mixture, necessitated a lower earth-to-water ratio. Additionally, swelling clays (bentonite and vermiculite) required more water to achieve equivalent flowability when compared to non-swelling clays (kaolinite and mica). Quantitatively, we set a plastic viscosity limit of 10 Pa·s as the criterion for water content adjustment, corresponding to ~2200 Pa at 220 s⁻¹, near the rheometer limit; higher viscosities are also impractical for extrusion-based 3D printing.

Four series were designed in the study to systematically examine these variables:

Series I: This series investigated the effect of SA concentration on the rheological performance of clay-sand suspensions. Biopolymer content was adjusted based on the water content (water-based design), as SA, being non-binding, remains in the solution rather than adsorbing onto clay surfaces. Despite variations in the earth-to-water ratio across different clay systems, the transition point for SA concentration consistently occurred at 0.12% biopolymer concentration (biopolymer-to-water, w/w) (Fig. 3), supporting the appropriateness of water-based biopolymer design for SA.

Series II: This series focused on the effect of LBG concentrations on the rheology of clay-sand suspensions, also using a water-based design strategy.

Series III: As LBG is a binding biopolymer, its interaction with clay surfaces necessitated additional considerations. To ensure the observed rheological trends for LBG were not influenced by design limitations, this series used a clay-based design for LBG additions in bentonite and vermiculite systems. For kaolinite and mica systems, a clay-to-water ratio of 1:1 was used, therefore no separate mixture adjustments were required.

Series IV: This series was designed specifically for mica systems, which exhibit strong settling behavior in the absence of polymers. In Series II, it was discovered that the mica-LBG samples exceeded the rheometer’s measurement range at LBG concentrations above 0.12%. Therefore, in Series IV, the earth-to-water ratio was reduced to 1:1, facilitating the testing concentrations up to 1.2% LBG.

#### Mix preparation

Kaolinite, Bentonite, Mica, and Sand were used as received. Due to its larger particle size, Vermiculite was ground using a LEJIEYIN Electric Grinder Mill (Model 550) for six min and sieved using a 1 mm sieve to ensure uniformity. The material preparation followed the methods described in our previous publications^22,56^. Initially, the clay and sand components were dry-mixed to achieve a homogeneous mixture. The biopolymers were dissolved separately in water by manually stirring with a spoon for one minute. This was followed by thorough mixing with a Hamilton Beach hand mixer (Model 62637) for an additional two min to ensure the biopolymers were fully dispersed. The prepared biopolymer solution was then combined with the clay-sand mixture and mixed for a total of three min. This mixing process included one minute with the hand mixer, one minute of manual stirring with a spoon, and another minute with the hand mixer to ensure a uniform and consistent mixture. The final prepared mixtures were promptly placed in either the rheometer’s cup for rheological testing or a syringe for 3D-printing. All experiments began seven min after the initial contact between water and the clay-sand mixture to ensure consistent testing conditions.

#### Rheological testing

The rheological behavior of the materials was systematically analyzed using a TA DHR-20 rheometer equipped with vane geometry (28 mm diameter) and a curved cup (34 mm diameter). The gap between vane and cup is 3 mm, more than five times bigger than the largest particle size of the materials as recommended by Mezger et al.^57^. To eliminate the effects of shear history and ensure a uniform starting condition, a pre-shear step was conducted at a constant shear rate of 220 s^−1^ for 300 s. Following this, the samples were allowed to rest for 300 s to equilibrate before proceeding to the main tests.

Yield stress was determined using a constant shear test. A steady shear rate of 1 s^−1^ was applied for 300 s, during which the shear stress was monitored. The peak shear stress recorded (yielding point) was identified as the yield stress of the mixture. The flow behavior of the mixtures was assessed using a multi-step shear rate increase protocol.

The shear rate was incrementally raised from 20 s^−1^ to 220 s^−1^ in steps of 20 s^−1^, with each step maintained for 30 s. The plastic viscosity and dynamic yield stress were calculated by fitting the downward flow curve to the Bingham Plastic model using the following equation:

1 $$\tau={\tau }_{0}+\mu \dot{\gamma }$$

where $\tau$ is the shear stress, ${\tau }_{0}$ is the dynamic yield stress, $\mu$ is the plastic viscosity, and $\dot{\gamma }$ is the shear rate.

The viscoelastic properties of the mixtures were examined through oscillatory strain amplitude sweeps and creep-recovery tests. For oscillatory strain amplitude sweep, the strain was varied logarithmically from 0.001% to 1000% at a constant frequency of 1 Hz, with data collected at 10 points per decade. This test identified the linear viscoelastic region (LVR) and the transition to non-linear behavior. In creep and creep-recovery tests, samples were subjected to a constant shear stress for 600 s. The applied shear stress for each mixture was carefully selected to remain below its yield stress, as summarized in Supplementary Table S3.2. The creep compliance, $J\left(t\right)$, was calculated using the following Eq. (2):

2 $$J\left(t\right)=\frac{\gamma (t)}{{\tau }_{c}}$$

Where, $\gamma (t)$ is the time-dependent strain and ${\tau }_{c}$ is the applied constant stress.

#### Small-scale print mixture proportions

Mesoscale 3D-printing experiments were designed to bridge the gap between rheological findings and large-scale printing applications. These experiments utilized engineered earth and natural earth material. The engineered earth was designed to replicate the composition and the particle size distribution of natural earth (Supplementary Fig. 10, Supplementary Table S3.3). As the natural earth contained predominantly fine sand particles, Ottawa Sand F-75 was utilized (165-210 μm). Since chlorite clay, accounting for 9% of the natural earth composition (Supplementary Fig. 10f), was outside of the minerals explored for this study, it was replaced with 4.5% mica and 4.5% kaolinite—selected for their close chemical properties to chlorite clay. The engineered earth, therefore, contained: 45.53% Ottawa Sand F-75, Kaolinite 21.51%, Bentonite 4.36%, Vermiculite 5.45%, and Mica 23.15% by weight. This engineered earth served as an intermediate material system, offering a controlled yet representative platform for testing and optimization prior to transitioning to natural earthen materials. As shown in Supplementary Table S3.3, there were three series of mixtures for mesoscale printing. This section focused on two primary aspects: the effects of biopolymer concentrations and the impact of varying solid content on the printability of the engineered and natural earth.

Series I: This series investigated the effects of different concentrations of LBG and SA on the printability of engineered earth. Since LBG and SA have two contrasting effects on material properties, different earth-to-water ratios were selected for the two groups. SA, which acts as a dispersant, requires stiffer mixtures at a higher earth-to-water ratio to emphasize its significant impact on flowability. Conversely, LBG, which increases stiffness, necessitated more flowable mixtures at a lower earth-to-water ratio to highlight its effect.

Series II: This series examined the influence of varying earth-to-water ratios on the buildability of engineered earth.

Series III: This series explored the effect of a 0.12% SA addition on the printability window of natural earth. This step extended the findings from an engineered earth to a natural earth system, assessing whether the addition of SA facilitates the continuous and smooth printing of mixture with higher solid content.

#### Small-scale printing

To evaluate the 3D-printability of the prepared mixtures (Supplementary Table S3.3), cylindrical samples (25.4 mm in diameter and height) were printed using a custom-designed syringe gantry printer, as detailed in ref. ^58^ (Supplementary Fig. 10j). This printer was equipped with a 60 ml syringe and a nozzle with a diameter of 2.4 mm. The samples were extruded at a constant rate of 40 mm/s. The printability of the mixtures was then classified into three categories based on the printing outcome: (1) Too-flowable: The material was unable to retain its shape or presents a significant yielding behavior, (2) Printable: The material can continuously and smoothly extrude from the nozzle and maintain its shape and dimensions, (3) Unprintable: The material failed to extrude consistently or blocked the nozzle.

#### Compressive strength measurement

Cylindrical specimens produced by small-scale 3D-printing were used for compressive strength testing. After printing, samples were cured under ambient conditions (23 ± 2 °C, 30 ± 5% relative humidity) for 7 days. Prior to testing, the ends of the samples were capped with gypsum to ensure flat and uniform loading surfaces. Compression tests were conducted using an MTS 7 kN universal testing machine under displacement control at a rate of 0.5 mm/min.

#### Drying shrinkage

Cylindrical specimens fabricated by small-scale 3D printing were also used to evaluate drying shrinkage. The initial height of each sample was measured immediately after printing, with measurement locations marked to ensure consistency. Following 7 days of ambient curing (23 ± 2 °C, 30 ± 5% relative humidity), the same marked positions were re-measured on the fully dried samples, and the drying shrinkage was recorded accordingly.

### Macroscale methods

#### Mix preparation

Two mix-designs were tested; the first using the parameters optimized at the micro and mesoscales: SA 0.12 % (biopolymer-to-water, w/w), 3:1 earth-to-water, here called SA 0.12. The second was a control, with only earth and water, at a 3:1 earth-to-water ratio, here called Control. The constituents were mixed with a planetary stand mixer (Avantco MX10H 10 Qt. Planetary Stand Mixer, USA). Initially, the water and biopolymer were mixed for 20 min. Subsequently, the natural earth was added, and the mixture was blended for an additional 30 min.

#### Printer setup

In this study, a WASP 40100 printer (WASP S.R.L., Italy) was selected as it is typically used to generate construction elements at the macroscale. The g-code script was generated in Rhinoceros 7 and Grasshopper environment (Rhinoceros 7, Robert McNeel & Associates). Printing speed and flow multiplier are defined in the G-code generator in the Rhino-Grasshopper environment. The air pressure is a parameter to be set on the 3D printer itself. The nozzle size selected was the biggest available with this model, namely, 8 mm, and the layer height was kept constant as 4 mm, i.e. half of the nozzle size^59^. Printing speed and air pressure were tested with a cylindrical specimen of 40 mm in diameter and 16 mm in height^60^. Overhang angles of 70°, 60°, and 50° were also assessed in 3D-printed elements of 52 mm height^61,62^. To conclude, a final demonstrator measuring 105 mm in height and 105 mm by 105 mm in length and width was printed to showcase the possibility of the technology to produce complex architected geometries (Supplementary Fig. 10k).

## Supplementary information

Supplementary Information

Transparent Peer Review file

## Source data

Source Data

## Data Availability

The data supporting the findings of this study are available within the paper and its Supplementary Information files. Source data are provided with this paper. Data are available from the corresponding author upon reasonable request.
