# Supplementary material for: Bio-inspired 3D-printed earthen materials and structures
Source: Nat Commun. 2026 Apr 18;17:5380. doi: 10.1038/s41467-026-71885-z (PMC13275924; doi:10.1038/s41467-026-71885-z)
Supplement: Supplementary file 1 — Supplementary Information [file 41467_2026_71885_MOESM1_ESM.pdf]

Supplementary Information

Bio-inspired 3D-printed Earthen Materials and Structures

Samuel J. Armistead[1]†[*], **Yierfan Maierdan[2]†**, **Olga B. Carcassi[3]†**, Rebecca A. Mikofsky[4], Shiho Kawashima[2], Lola Ben-Alon[3], Wil V. Srubar III[1,4]

[1]*Department of Civil, Environmental, and Architectural Engineering, University of Colorado Boulder, Boulder, CO 80309, USA*

[2]*Department of Civil Engineering and Engineering Mechanics, Columbia University, New York, NY 10027, USA*

[3]*Graduate School of Architecture, Planning and Preservation, Columbia University, New York, NY 10027, USA*

[4]*Materials Science and Engineering Program, University of Colorado Boulder, Boulder, CO 80309, USA*

†These authors contributed equally: Samuel J. Armistead, Yierfan Maierdan, Olga B. Carcassi

*To whom all correspondence should be addressed: samuel.armistead@colorado.edu

List of Supplementary

1. **Supplementary Discussion**

   1.1. Microscale

   1.1.1. Sand and Clay Chemistry

   1.1.2. Biopolymer Chemistry

   1.2. Mesoscale

   1.2.1. Clay Rheology

   1.3. Macroscale

   1.3.1. Natural Earth Characterization

2. **Supplementary Figures**

   Supplementary Figure 1. Sand & Clay Characterization

   Supplementary Figure 2. Sand & Clay SEM

   Supplementary Figure 3. Clay Illustration & XRF

   Supplementary Figure 4. pH & Electrical Conductivity

   Supplementary Figure 5. Biopolymer Characterization & Illustration

   Supplementary Figure 6. ATR-FTIR of Biopolymer-Mineral Binding Characterization

   Supplementary Figure 7. Binding Model Illustrations

   Supplementary Figure 8. Effect of SA and LBG Concentrations on Clay-Sand Rheology

   Supplementary Figure 9. Effect of LBG:Clay on Clay-Sand Rheology

   Supplementary Figure 10. Natural Earth Characterization & 3D-printing Protocol

   Supplementary Figure 11. Printed Natural Earth Shrinkage & Compressive Strength.

3. **Supplementary Methods**

   Table S3.1. Mix Proportions for Rheological Characterizations

   Table S3.2. The Constant Shear Stress Applied for Creep Tests

   Table S3.3. Mixture Proportions for Meso-Scale Printing

1. Supplementary Discussion

1.1. Microscale Discussion

1.1.1. Sand and Clay Chemistry

Sand particles have a rounded shape with a surface chemistry of 50% hydrophobic siloxane groups and 50% negatively charged deprotonated silanol groups (Supplementary Fig. 7e)[32]. Clays, in contrast, are composed of platelets with two distinct chemistries: basal planes and edge planes. Bentonite, characterized by the lowest platelet interlayer charge (-0.36) (Supplementary Fig. 3b), exhibits weak interactions between its interlayer cations and predominantly hydrophobic siloxane basal planes (Supplementary Fig. 3a)[33, 34]. When exposed to water, these weak interactions lead to platelet expansion, driven by interlayer cation hydration, which is known as crystalline swelling[35]. Large differences in ion concentration between water close to the clay surface and pore water then results in ion release into solution, known as osmotic swelling (Supplementary Fig. 3a)[35]. These phenomena result in an increase in pH and a slight rise in electrical conductivity due to the generation of metal ions and hydroxide ions in the pore solution (Supplementary Fig. 4b).

In contrast, mica, with the highest interlayer charge (-0.87) resulting from extensive isomorphic substitution (Supplementary Fig. 3b), forms strong interactions between its highly charged siloxane and interlayer cations (Supplementary Fig. 3a)[31]. This leads to the formation of stable tactoid structures (Supplementary Fig. 3a) with minimal changes to pore solution chemistry (Supplementary Fig. 4d)[34]. Vermiculite, possessing an intermediate interlayer charge (-0.78), strikes a balance between swelling, tactoid retention, and moderate changes in pore solution chemistry (Supplementary Fig. 3a, 3b, 4c). Kaolinite features a neutrally charged alumina face and a hydrophobic siloxane face (Supplementary Fig. 3a), with limited isomorphic substitution resulting in a small number of negatively charged groups on the siloxane face. This surface chemistry facilitates strong hydrogen bonding between the predominantly neutrally charged

siloxane and alumina faces, leading to stable tactoid structures (Supplementary Fig. 3a)[33, 34, 36]. Like mica, kaolinite induces minimal changes in pore solution chemistry (Supplementary Fig. 4a). Edge group behavior varies significantly across clays under different pore solution conditions. Kaolinite, at neutral pH (pH 7), exhibits negatively charged edge groups (Supplementary Fig. 3a). In 2:1 clays, increased isomorphic substitution raises the pKa of edge hydroxyl groups, making them less acidic[37]. Bentonite, capable of creating a high pore solution pH (pH 9), also features negatively charged edge groups due to this elevated pH environment (Supplementary Fig. 3a, 4b). Conversely, due to a high level of isomorphic substitution, vermiculite (pH 8) and mica (pH 7) exhibit positively charged edge groups under their respective pore conditions (Supplementary Fig 3a, 4c, d)[37].

The interplay between edge and basal plane chemistries is crucial in determining the resulting assembly-level interactions. In kaolinite, the hydrogen-bond driven stacking of platelets into tactoids creates large negatively charged edge groups, which can interact favorably with alumina face groups, resulting in the formation of a face-to-edge microfabric structures (Supplementary Fig. 3a)[36]. Bentonite, due to weak interlayer interactions and negatively charged edge groups, exhibits minimal tactoid formation or interaction, preventing the development of an organized microfabric (Supplementary Fig. 3a). Vermiculite, due to its intermediate interlayer charge, forms smaller tactoid structures. Consequently, despite possessing positively charged edge groups and negatively charged basal planes, it fails to achieve the edge dimensions necessary for the development of face-to-edge interactions and an ordered microfabric structure (Supplementary Fig. 3a). In contrast, mica forms larger, stable tactoid structures that enable face-to-edge interactions[38]. These interactions are facilitated by its positively charged edge groups and highly negatively charged basal planes (Supplementary Fig. 3a), resulting in a face-to-edge microfabric structure.

1.1.2. Biopolymer Chemistry

Nature demonstrates extraordinary precision in optimizing biopolymers at the atomic level. Polysaccharides are composed of individual monosaccharide units, which are carbon-based ring structures featuring attached hydroxyl (-OH) groups[45]. The spatial arrangement of hydroxyl groups, either in axial or equatorial positions, plays a crucial role in determining their chemical properties and reactivity[46]. For instance, glucose, the most common monosaccharide building block, has hydroxyl groups predominantly in equatorial positions, which promotes hydrophilicity. In contrast, galactose has a single axial hydroxyl group at C-4, while mannose features an axial hydroxyl group at C-2. These small structural variations introduce distinct steric and hydrogen-bonding characteristics, resulting in reduced solubility of galactose and mannose compared to glucose (Supplementary Fig. 5b)[46].

Monosaccharides are linked through glycosidic bonds—ether linkages formed between hydroxyl groups—to produce polysaccharide biopolymers. The formation of long chains and the loss of free hydroxyl groups during backbone or side-chain assembly significantly alter the chemical properties of the resulting polysaccharide. For example, while glucose is highly hydrophilic due to its free hydroxyl groups, cellulose, a polysaccharide composed entirely of glucose subunits, is insoluble. This insolubility arises from the strong hydrogen-bonding network formed between the equatorial hydroxyl groups of glucose groups on adjacent cellulose chains, creating a rigid and tightly packed structure[47].

Similarly, galactomannans, which consist of a mannose backbone with galactose side chains, exhibit unique properties driven by their specific structural arrangement (Supplementary Fig. 5b, c). The backbone formation primarily involves the C-1 and C-4 hydroxyl groups of mannose, while the spatial orientation of the remaining mannose hydroxyl groups at C-2 and C-5, contributes significantly to the development of hydrophobic characteristics (Supplementary Fig. 5b, c)[48]. In contrast, the galactose side chains, with their axial hydroxyl group at C-4 and their

connection to the mannose backbone at C-1, are highly hydrophilic, enhancing solubility and hydration (Supplementary Fig. 5b, c). Therefore guar gum, with a 1:2 galactose-to-mannose ratio, exhibits greater hydrophilic properties, whereas cassia gum, with a 1:5 galactose-to-mannose ratio, exhibits greater hydrophobic properties (Supplementary Fig. 5b, c).

Nature further optimizes polysaccharide stabilizers through functionalization, particularly at the C-6 position. This position can be modified to introduce chemical groups such as carboxyl groups, acetyl groups, or ketal linkages (Supplementary Fig. 5b, c), adding structural complexity and diversity. Sodium alginate exemplifies this, with its negatively charged glucuronic acid and mannuronic acid units (Supplementary Fig. 5b, c), which both contribute to its high solubility[49].

Further structural diversity is achieved through additional modifications, as seen in xanthan gum. Xanthan gum features a glucose backbone with a trisaccharide side chain composed of a mannose unit functionalized with hydrophobic acetyl groups, a negatively charged glucuronic acid unit, and another mannose unit bearing a negatively charged pyruvate group (Supplementary Fig. 5b, c). These functional groups contribute to xanthan gum's distinctive properties. Unlike galactomannans and alginates, which typically adopt random coil conformations in solution, xanthan gum forms an antiparallel double-helix tertiary structure. In this configuration, the hydrophobic acetyl groups are sequestered within the helix, enhancing stability in aqueous environments[50]. This structural arrangement causes extensive intramolecular and intermolecular interactions, leading to the formation of larger molecular assemblies (Supplementary Fig 5b, c).

This precise optimization of polysaccharide molecular chemical structures underscores nature’s remarkable ability to engineer biopolymers for diverse and specialized functions. However, these subtle molecular variations have yet to be fully optimized for stabilizing earth materials in 3D-printing—a key objective of this manuscript.

1.2. Mesoscale Discussion

1.2.1. Clay Rheology

Among the 2:1 clays, bentonite-sand system exhibited the lowest plastic viscosity yet the highest yield stress, while the mica-sand system demonstrated the opposite trend, with vermiculite occupying an intermediate position (Fig. 3e). These disparities can be attributed to the inherent properties of these clays, primarily their interlayer charge and resulting swelling ability.

Bentonite is characterized by a low interlayer charge (-0.36) (Supplementary Fig. 3b) with multiple water layers, which is also referred to as crystalline swelling[31, 51]. This swelling behavior results in the formation of a three-dimensional gel structure with multiple bonding points, leading to a higher yield stress compared to non-swelling mica or intermediate-swelling vermiculite[52, 53] (Fig. 3e). As the interlayer charge increases from bentonite (-0.36) to vermiculite (-0.78) to mica (-0.87), the microstructure of these clays responds differently to flow conditions. Bentonite, due to its low layer charge and weaker interlayer interactions, once yielded, forms a microstructure made up of exfoliated sheets which can more easily align with the flow direction, resulting in lower viscosity (Fig. 3e-h, control groups). In contrast, mica's strong interlayer charges hinder the breakdown of its microstructure into such small particles, leading to a higher viscosity (Fig. 3e-h, control groups). Vermiculite, with intermediate properties, exhibits intermediate behavior. Kaolinite, a non-swelling clay, shares similarities with mica. However, its smaller particle size (Fig. 2d) results in a more densely packed network with increased contact points, contributing to a higher yield stress at similar viscosities (Fig. 3e-h, control groups).

From a rheological standpoint, swelling clays such as bentonite and vermiculite are the most suitable for 3D-printing, offering an optimal balance of low viscosity and high yield stress. Kaolinite provides moderate printability, while mica is the least favorable due to its high viscosity and comparatively lower yield stress. Therefore, the presence of swelling clays in natural earth may enhance printability. However, their high water demand introduces challenges such as

increased drying shrinkage during dehydration. While yield stress and buildability can be improved by increasing the solid volume fraction and increasing swelling clay content, the critical challenge lies in optimizing the solid volume fraction to minimize drying shrinkage and enhance the mechanical strength of printable materials. The pronounced dispersion effect of SA—particularly at lower concentrations (e.g., 0.12%)—facilitates minimizing the water demand for swelling and non-swelling clay systems, reinforcing the promise of SA for further exploration.

1.3. Macroscale Discussion

1.3.1 Natural Earth Characterization

To support the quantitative mineralogical characterization of heterogeneous earth material, first, a qualitative XRD protocol was carried out (Supplementary Fig. 10d). The untreated natural earth samples had initial peaks at ~15, 10, 7.2, 5 angstroms (~5.9, 8.8, 12.3 and 17.7 degrees 2θ respectively). In ethylene glycol treated natural earth, the ~15Å peak partially shifted to 17Å and partially remained at ~14Å, while the 10, 7, and 5Å peaks exhibited no change. The 300°C treated natural earth had a small peak at ~14Å and peaks at 10, 7, and 5Å. After additional heating to 550°C, the 10 and 5Å peaks remained while the 7Å peak was mostly destroyed. These shifts indicate the presence of montmorillonite (15→17Å), vermiculite and chlorite (15→14Å), micas (~10Å), and kaolinite (7Å).

2. Supplementary Figures

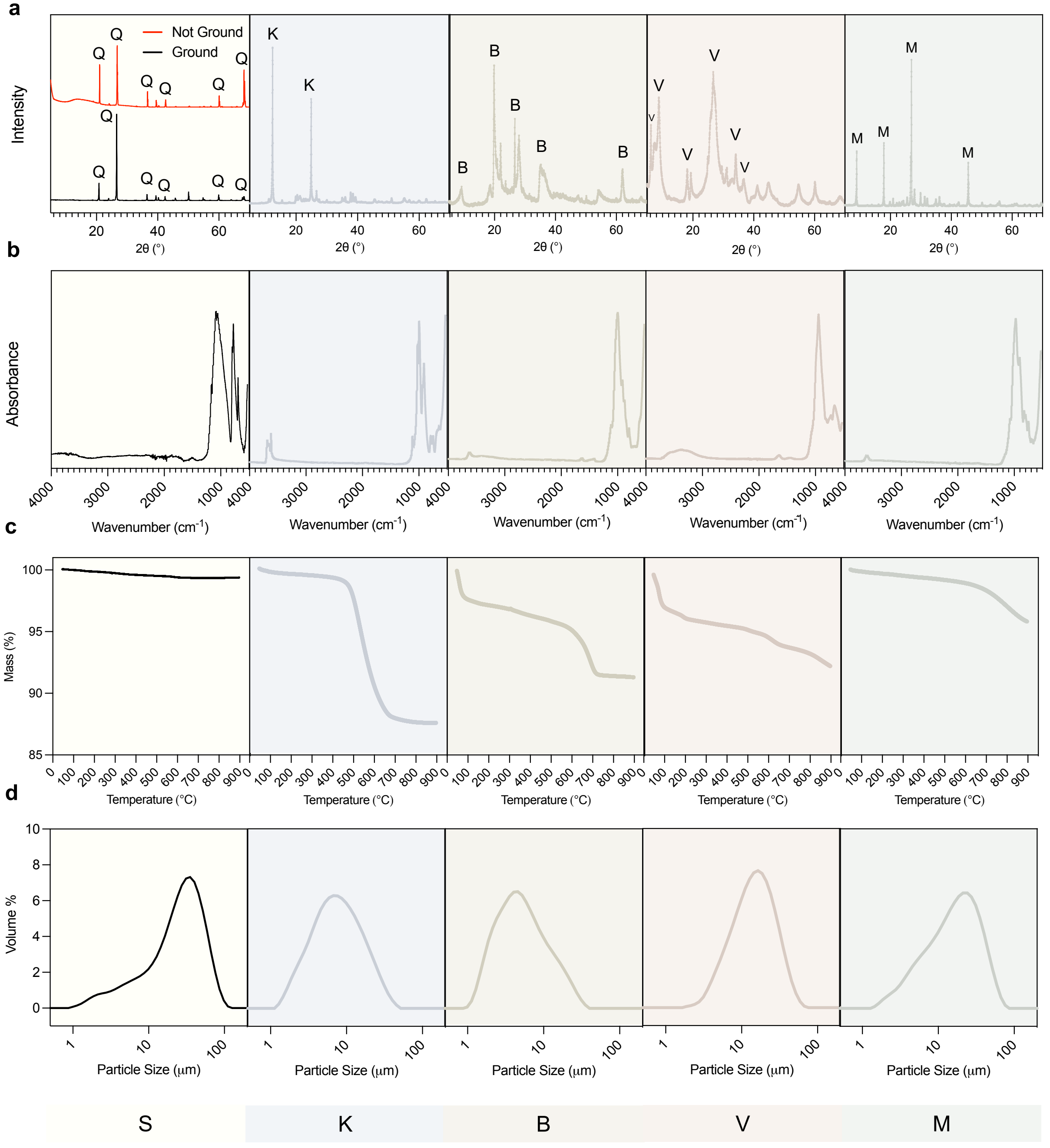

Supplementary Fig. 1 Sand & Clay Characterization. a, XRD, **b,** ATR-FTIR, **c,** TGA (mean, n=2)**, d,** PSA

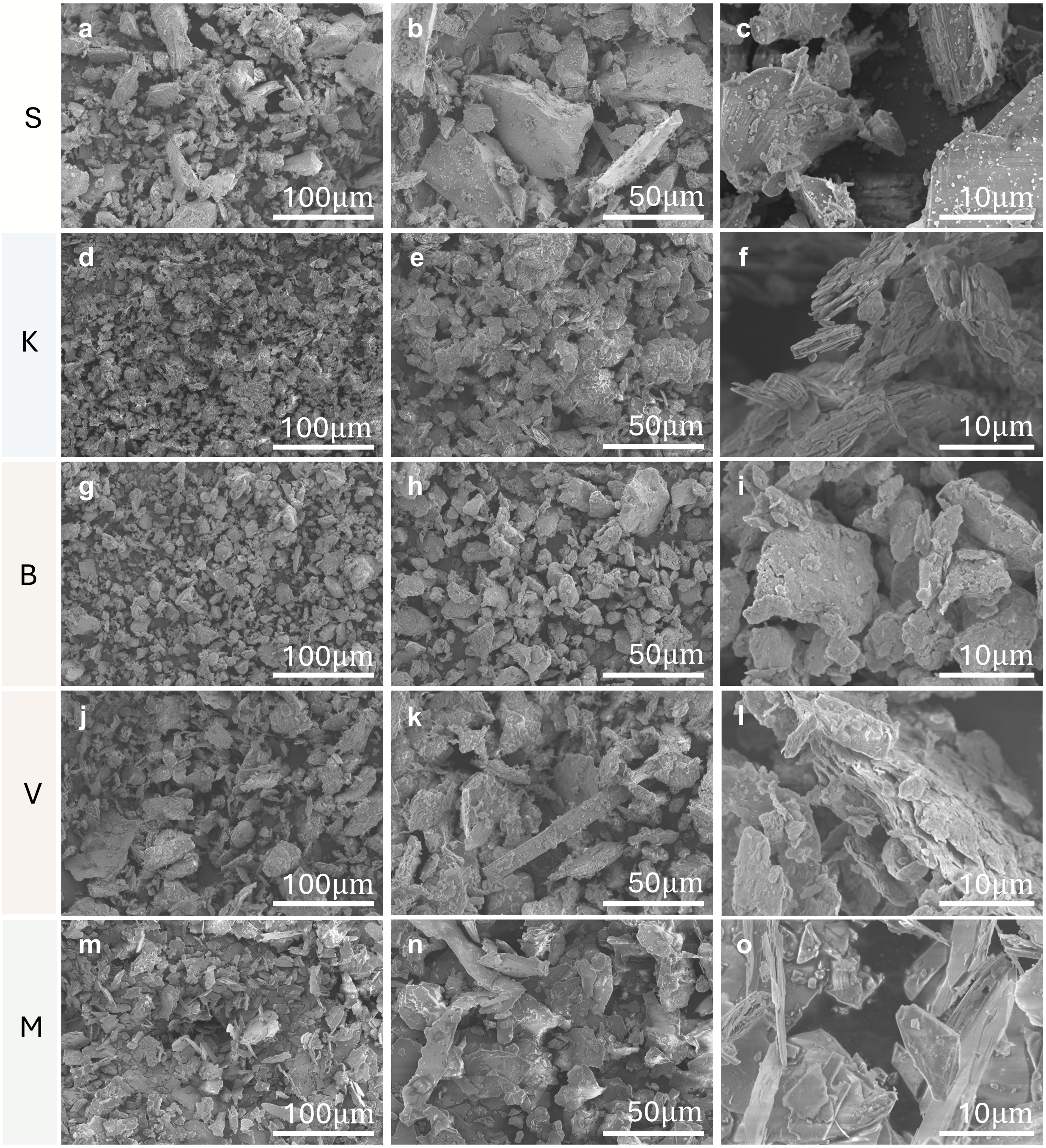

Supplementary Fig. 2 Sand & Clay SEM. a, b, c, Sand, **d, e, f**, Kaolinite, **g, h, i,** Bentonite, **j, k, l,** Vermiculite, **m, n, o,** Mica

a

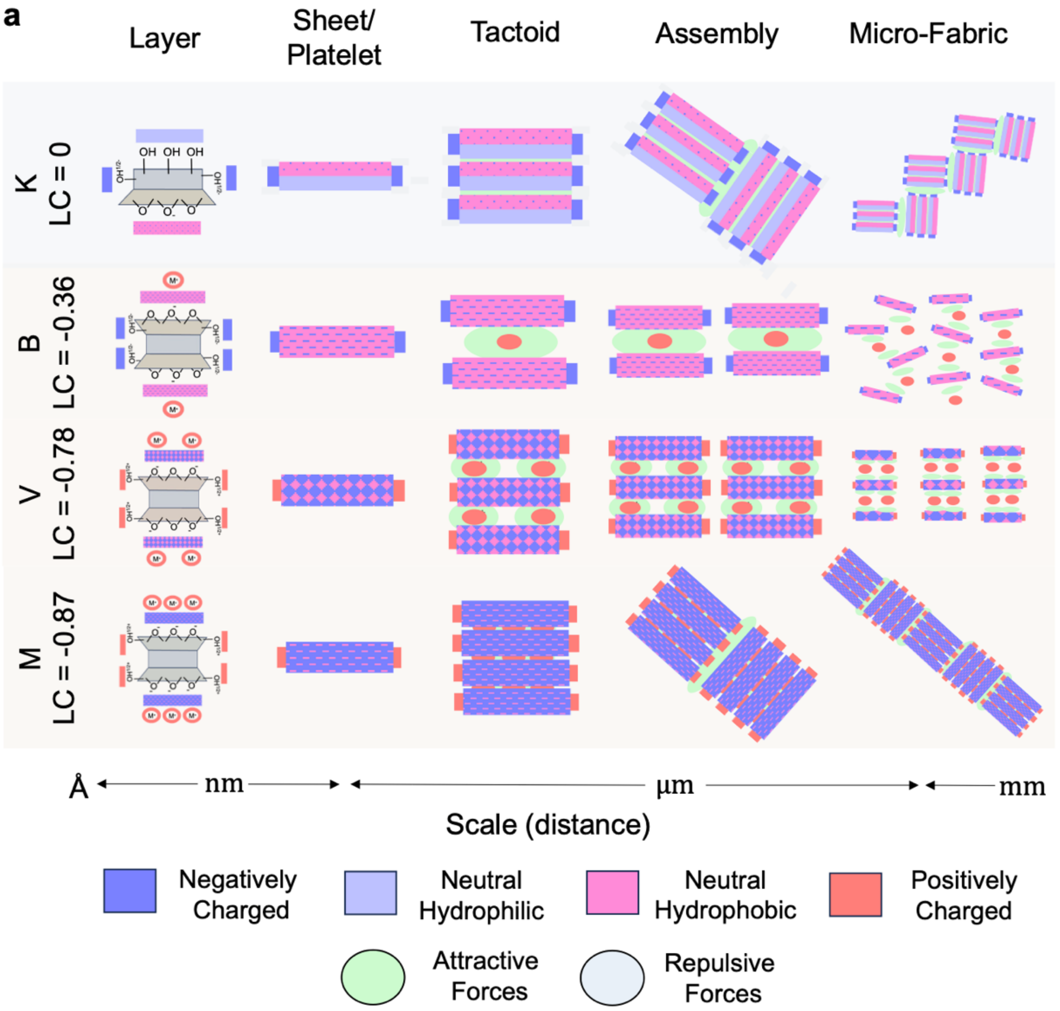

b

	Sand	Kaolin	Bentonite	Vermiculite	Mica
SiO_2	95.75	49.56	63.07	38.06	56.64
Al_2O_3	0.91	37.26	20.52	8.51	25.6
Fe_2O_3	0.02	0.44	3.79	9.69	2.63
CaO	0.03	0.07	1.13	3.58	0.07
MgO	0.05	0.24	2.6	21.51	2.45
Na_2O	0	0	2.06	0.1	1.13
K_2O	0	1.73	0.39	4.64	8.89
		Layer Charge	-0.36	-0.78	-0.87

Supplementary Fig. 3 Clay Illustration & XRF. a, Clay Illustration, LC – Layer Charge **b,** XRF of Clay & Sands, all concentrations are in weight percentage (%). Clay layer charge was determined via the structural formula method[54].

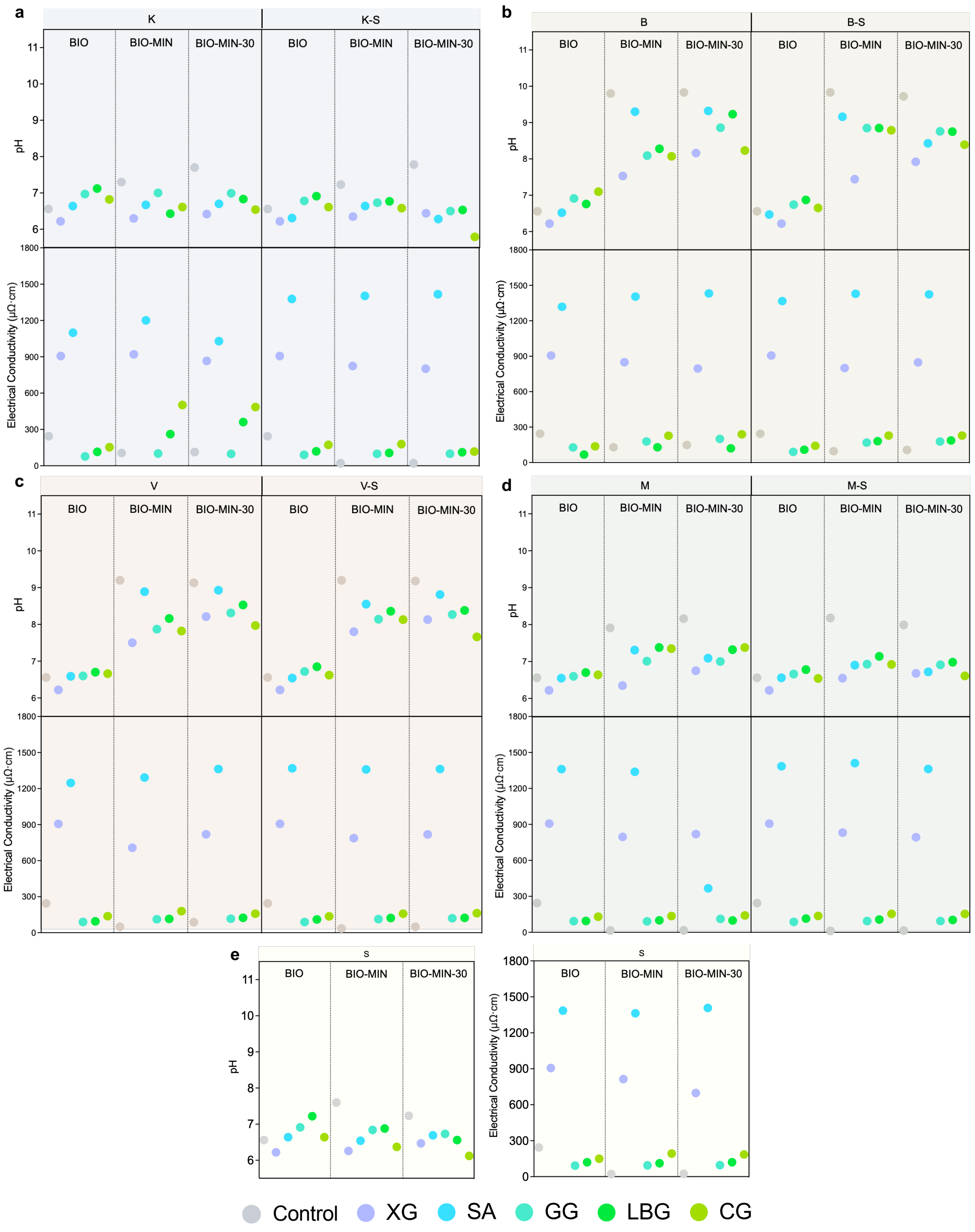

Supplementary Fig. 4 pH & Electrical Conductivity. pH and Electrical Conductivity throughout the biopolymer-mineral binding characterization process was taken, first at initial biopolymer solution (BIO), then after the addition of minerals and 10 minutes sonication (BIO-MIN), and finally after 30 minutes agitation (BiO-MIN-30): **a**, Kaolinite (K) and Kaolinite-Sand (K-S), **b**, Bentonite (B) and Bentonite-Sand (B-S), **c**, Vermiculite (V) and Vermiculite-Sand (V-S), **d**, Mica (M) and Mica-Sand (M-S), **e,** Sand (S).

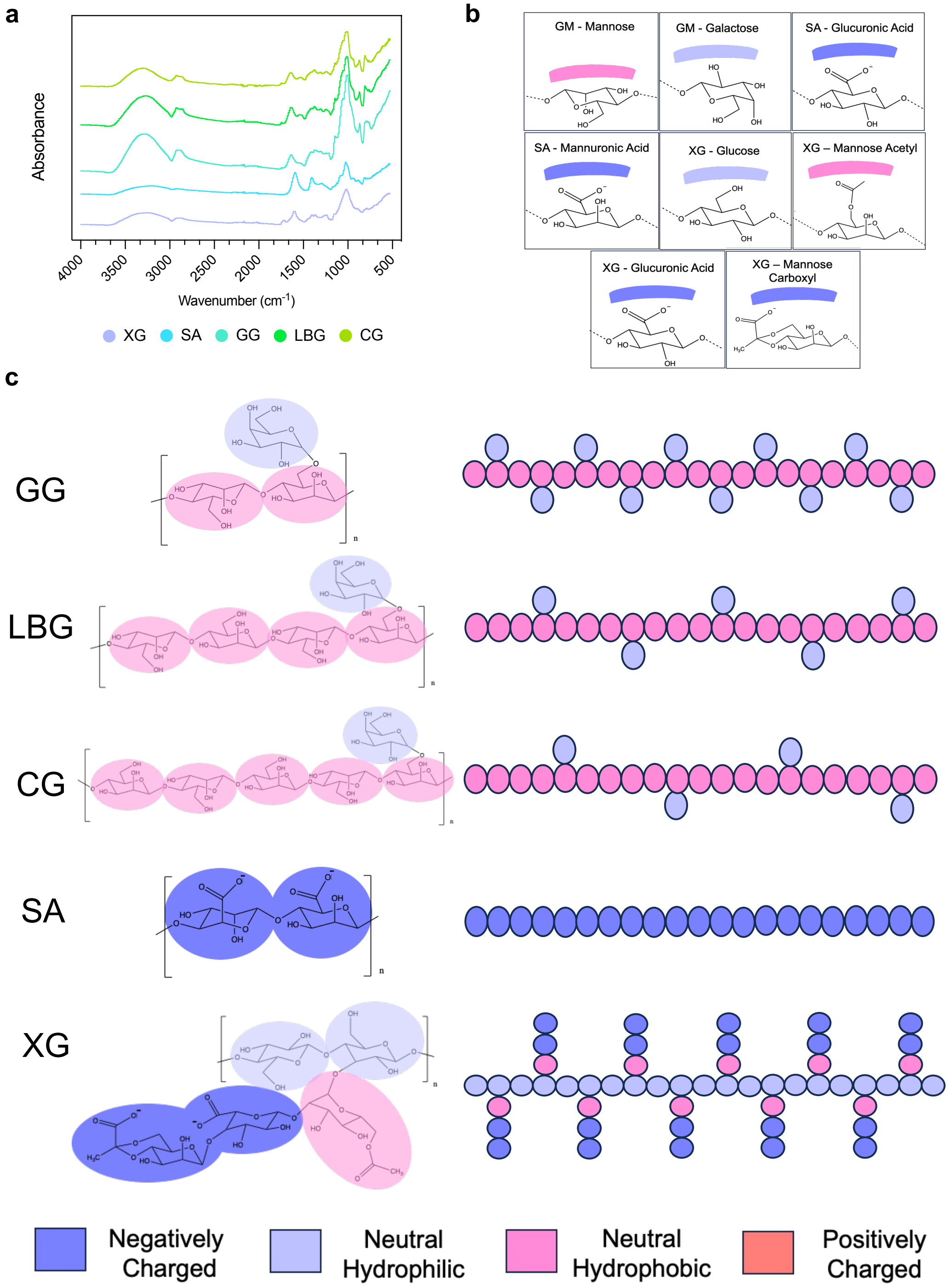

Supplementary Fig. 5 Biopolymer Characterization & Illustration. a, Biopolymer ATR-FTIR**, b,** Monosaccharide building blocks, **c,** Biopolymer repeat units and 20 unit backbone, highlighting the chemistry of subunits.

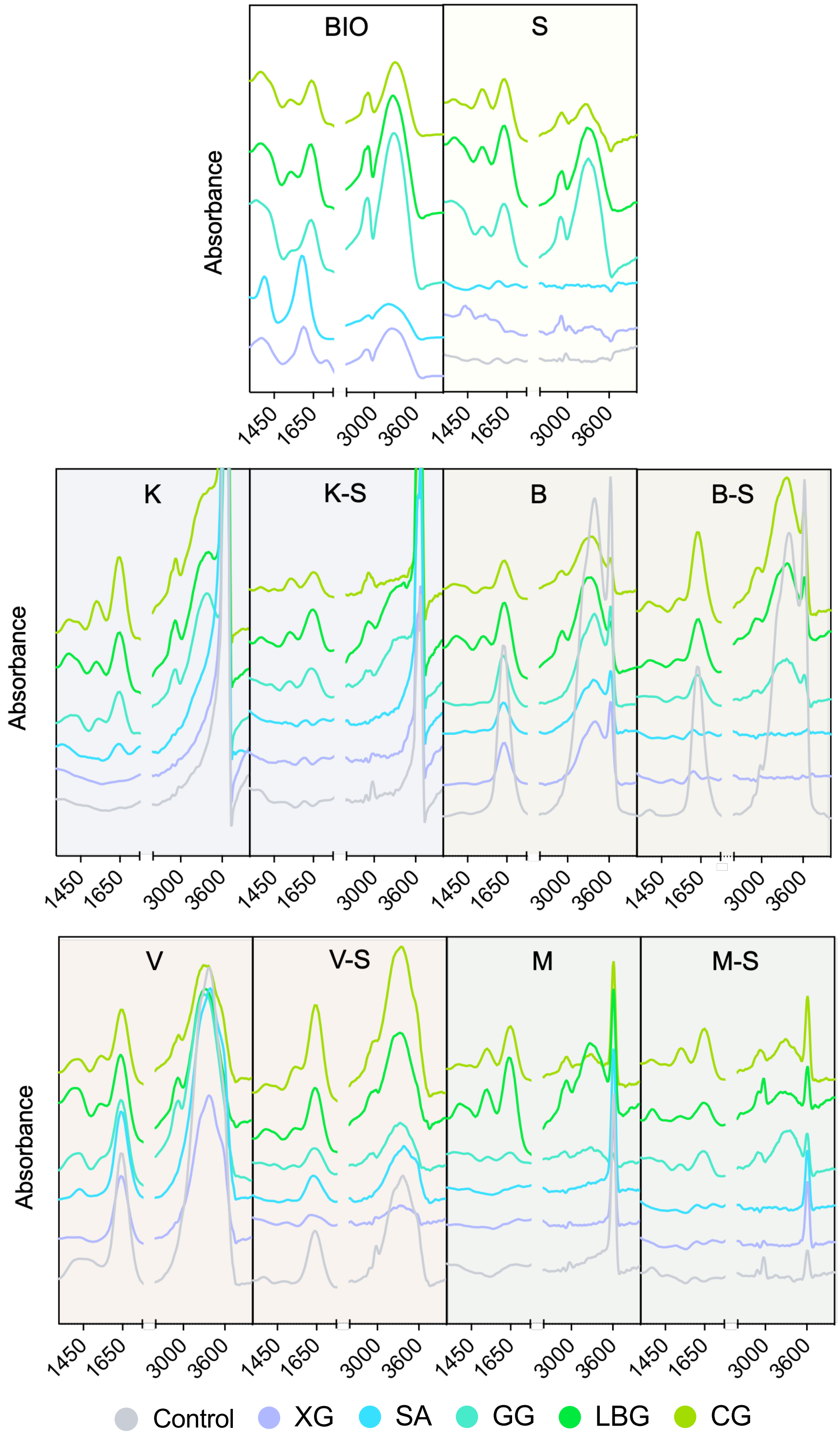

Supplementary Fig. 6 ATR-FTIR of Biopolymer-Mineral Binding Characterization. ATR-FTIR of biopolymer-mineral samples: BIO - Biopolymer Controls.

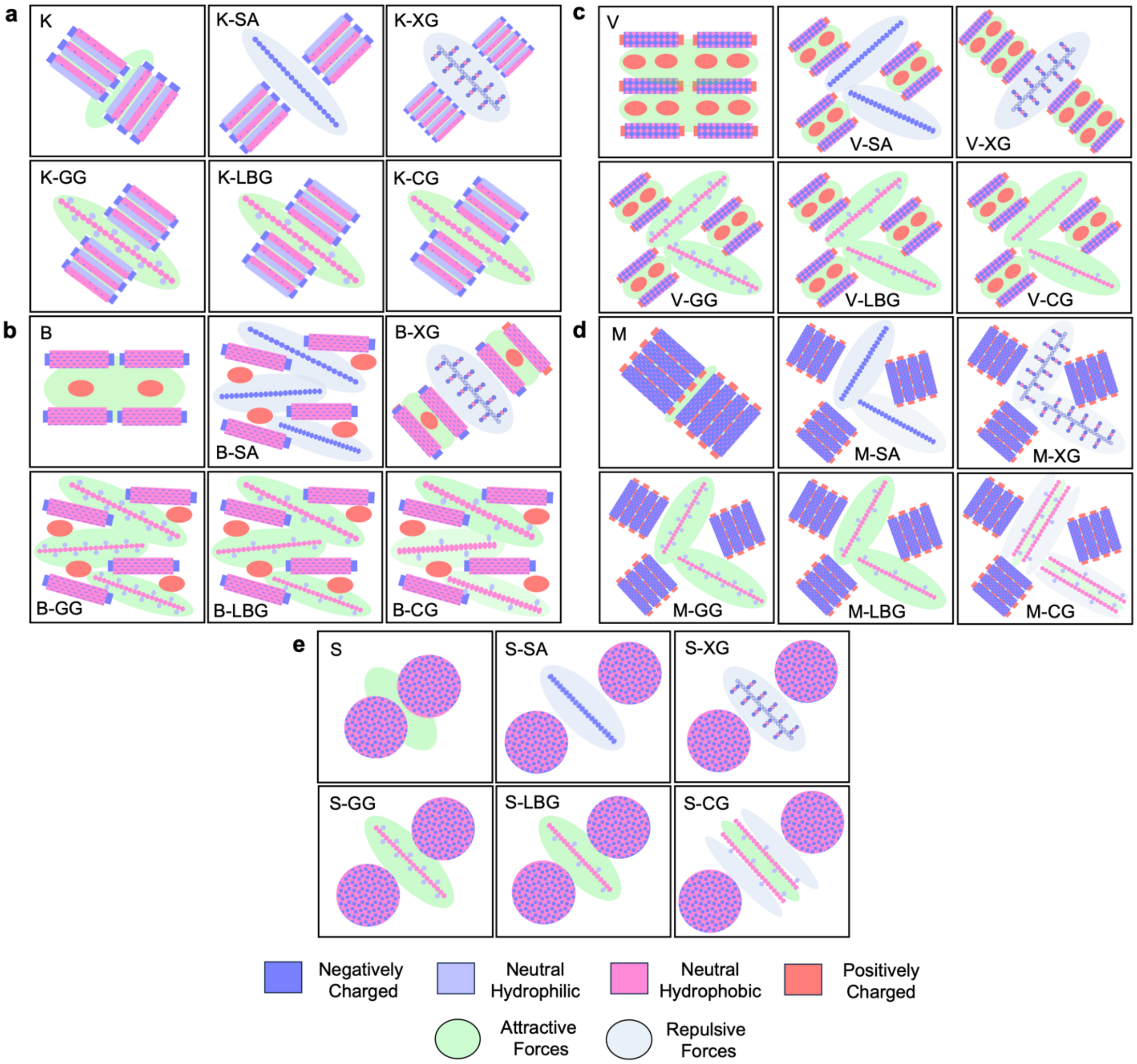

Supplementary Fig. 7 Binding Model Illustrations. Biopolymer-mineral interactions of SA, XG, GG, LBG and CG with **a**, K, **b**, B, **c**, V, **d**, M, **e**, S

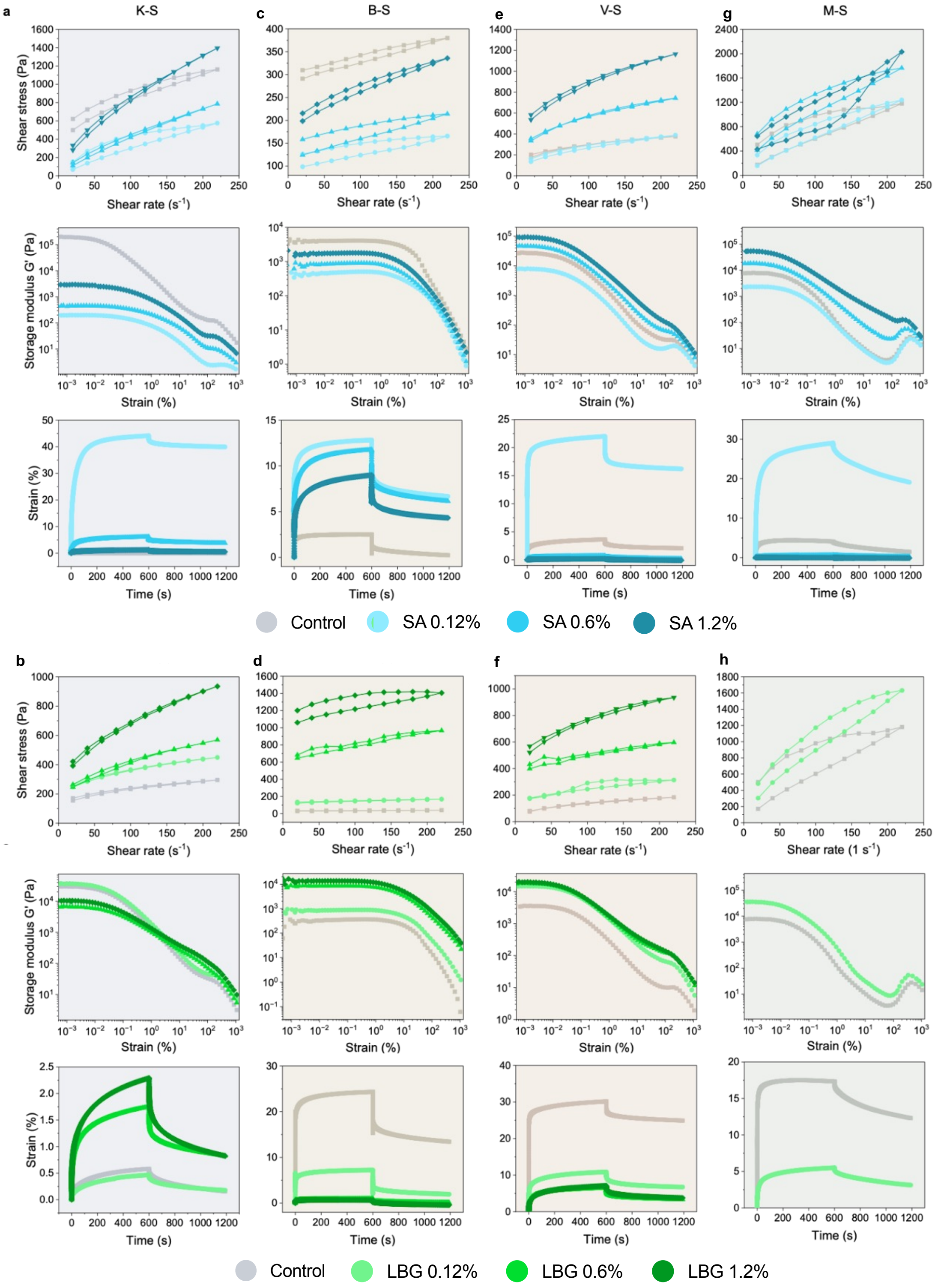

Supplementary Fig. 8 Rheological Characterization of the effect of SA and LBG Concentrations on Clay-Sand Systems (SERIES I, II). Influence of biopolymer concentration on: Flow curve (top); Strain amplitude sweep (storage modulus) (middle); and creep recovery (bottom) for: **a,** K-SA, **b,** K-LBG, **c,** B-SA, **d,** B-LBG, **e,** V-SA, **f,** V-LBG, **g,** M-SA, **h,** M-LBG.

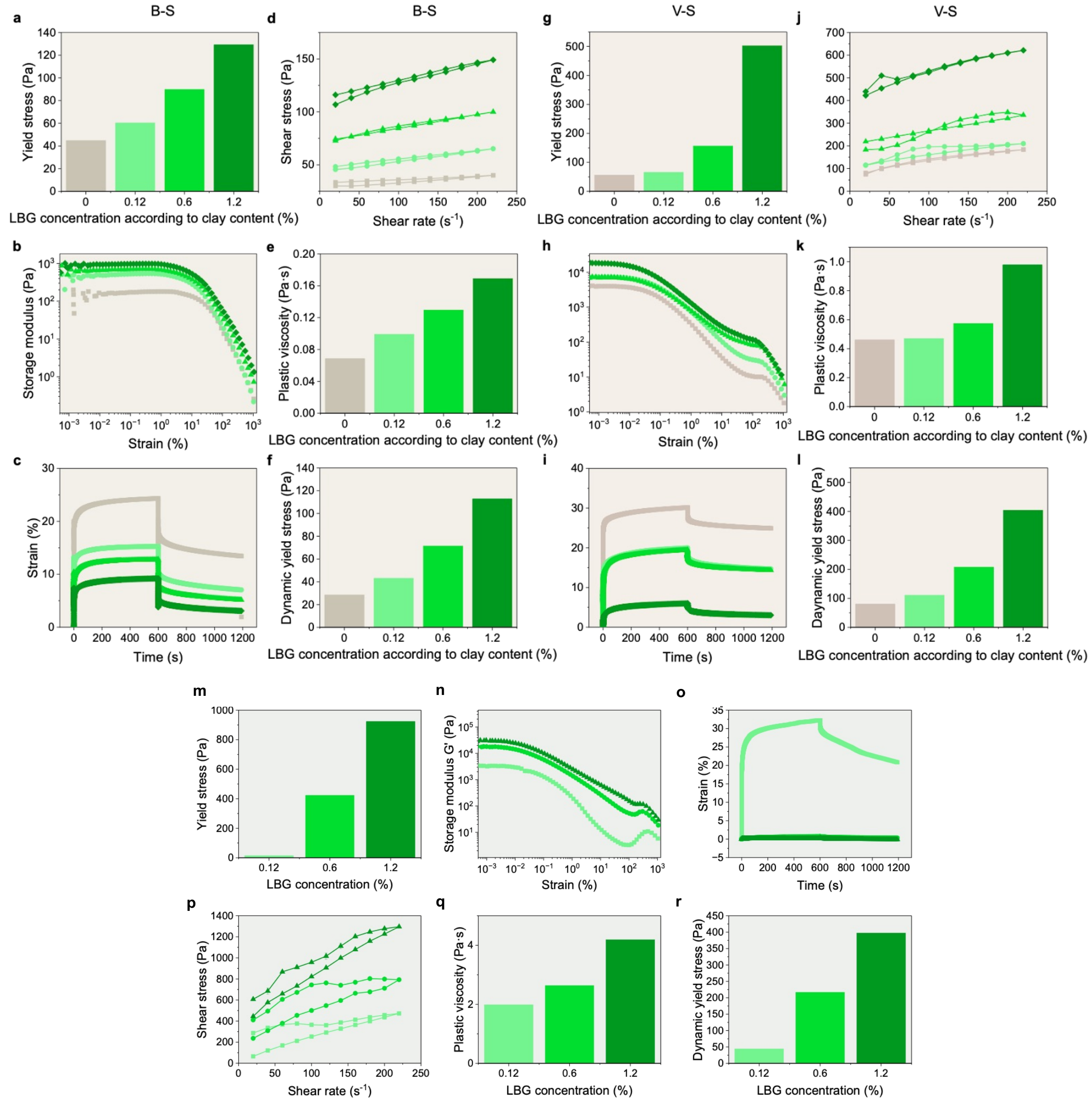

Supplementary Fig. 9 Rheological Characterization of Designing Biopolymer LBG Concentrations According to Clay Contents (Series III, IV). Influence of LBG concentration on bentonite-sand system with clay-to-water ratio 1/9: **a**, Yield stress **b**, Strain amplitude sweep (storage modulus) **c**, Creep and creep-recovery **d**, Flow curve **e**, Plastic viscosity **f**, Dynamic yield stress of bentonite-sand system. Influence of LBG concentration on vermiculite-sand system with clay-to-water ratio 1/2: **g**, Yield stress **h**, Strain amplitude sweep (storage `modulus) **i**, Creep and creep-recovery **j**, Flow curve **k**, Plastic viscosity **l**, Dynamic yield stress. Influence of LBG concentration on mica-sand with 1/1 clay-to-water ratio: **m**, Yield stress **n**, Strain amplitude sweep (storage modulus) **o**, Creep and creep-recovery **p**, Flow curve **q**, Plastic viscosity **r**, Dynamic yield stress.

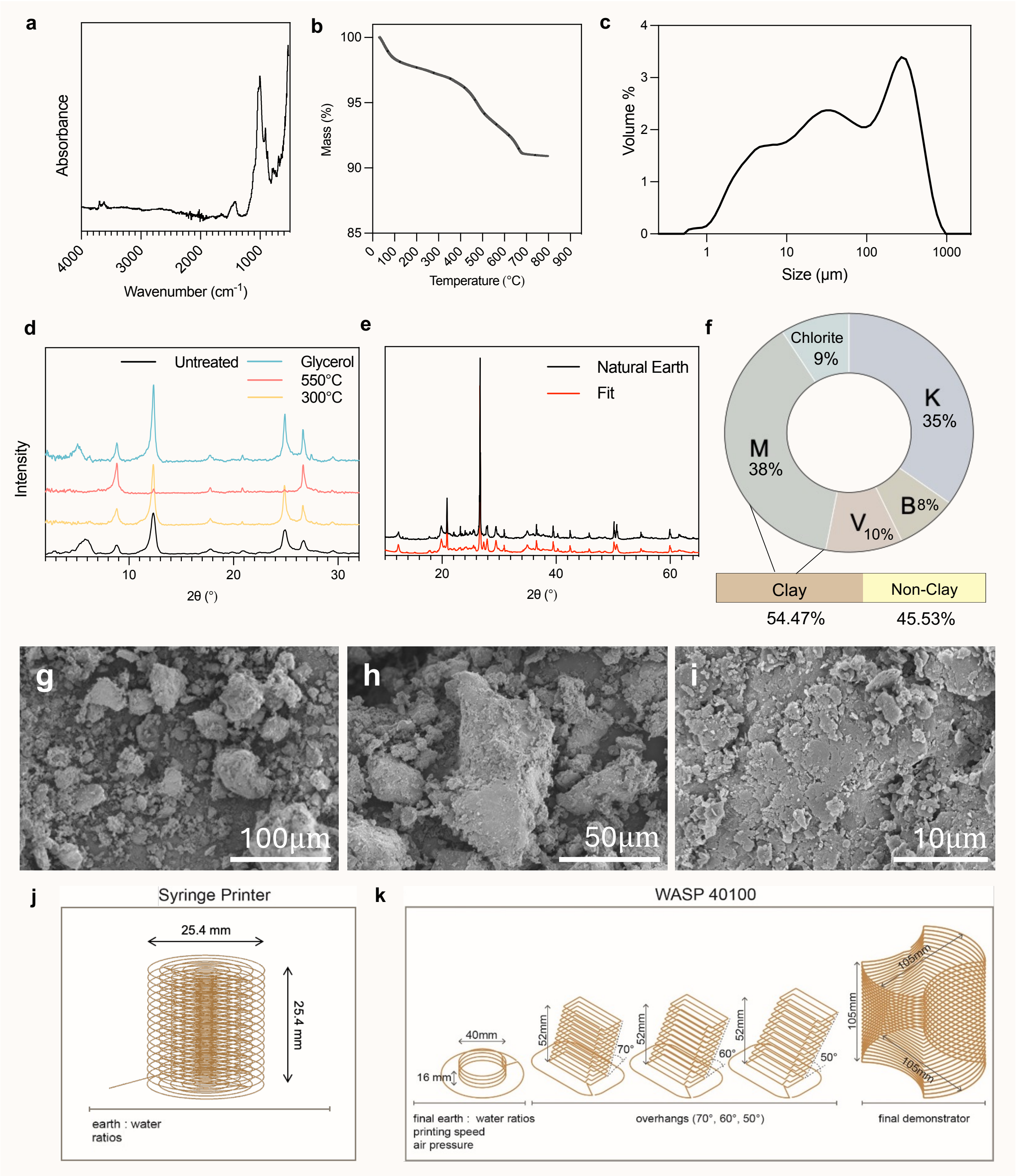

Supplementary Fig. 10 Natural Earth Characterization & 3D-printing Protocol. a, ATR-FTIR. **b,** TGA. **c,** PSA, **d,** qualitative XRD analysis, **e,** quantitative XRD analysis, **f,** Bar chart representing the clay and non-clay components and a pie chart representing the clay types in the clay portion based on qualitative and quantitative XRD analysis, **g, h, i,** SEM of natural earth, **j,** Printing path and geometry of mesoscale syringe printer **k**, Printing paths and geometries printed with the macroscale WASP 40100.

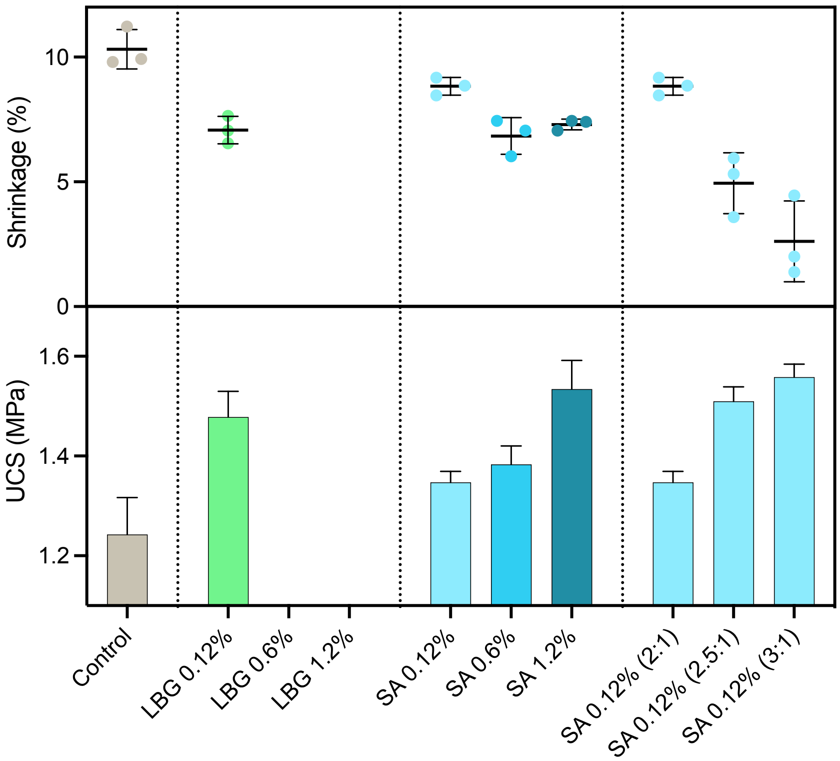

Supplementary Fig. 11 Syringe Printed Natural Earth Shrinkage & Compressive Strength. Linear shrinkage of printed samples measured via initial height (25.4mm) against final height of sample following curing. The addition of SA 0.12% enabled an increased earth:water ratio from 2:1 to 3:1 (indicated in parenthesis on figure) which facilitated a significant reduction in shrinkage versus the control from 10.31% to 2.61% and a statistically significant 25% increase in mean compressive strength increase from 1.24 MPa to 1.56 MPa (Welch's t-test, $p = 0.011$). The addition of LBG 0.6% and 1.2% resulted in a non-extrudable mixture, therefore no shrinkage or compressive strength could be measured. All data are presented as mean ± SD ($n = 3$).

3. Supplementary Methods

Table S3.1 Mix Proportions for Rheological Characterizations

Series	Groups	Mix ID	Kaolinite (g)	Bentonite (g)	Vermiculite (g)	Mica (g)	Sand (g)	Water (g)	SA (g)	LBG (g)	Water/clay ratio	Biopolymer/ water ratio	Biopolymer/ clay ratio
**Design biopolymer content according to water content**													
I	Kaolinite-sand-SA	K_SA_control	120	-	-	-	240	100	0		5/6	0	
		K_SA0.12							0.12			0.12	
		K_SA0.6							0.6			0.6	
		K_SA1.2							1.2			1.2	
	Bentonite-sand-SA	B_SA_control	-	14.3	-	-	28.6	100	0		7/1	0	
		B_SA0.12							0.12			0.12	
		B_SA0.6							0.6			0.6	
		B_SA1.2							1.2			1.2	
	Vermiculite-sand-SA	V_SA_control	-	-	60	-	120	100	0		5/3	0	
		V_SA0.12							0.12			0.12	
		V_SA0.6							0.6			0.6	
		V_SA1.2							1.2			1.2	
	Mica-sand-SA	M_SA_control	-	-	-	120	240	100	0		5/6	0	
		M_SA0.12							0.12			0.12	
		M_SA0.6							0.6			0.6	
		M_SA1.2							1.2			1.2	
II	Kaolinite-sand-LBG	K_L_control	100	-	-	-	200	100		0	5/5	0	
		K_L0.12								0.12		0.12	
		K_L0.6								0.6		0.6	
		K_L1.2								1.2		1.2	
	Bentonite-sand-LBG	B_L_control	-	11.1	-	-	22.2	100		0	9/1	0	
		B_L0.12								0.12		0.12	
		B_L0.6								0.6		0.6	
		B_L1.2								1.2		1.2	
	Vermiculite-sand-LBG	V_L_control	-	-	50	-	100	100		0	2/1	0	
		V_L0.12								0.12		0.12	
		V_L0.6								0.6		0.6	
		V_L1.2								1.2		1.2	
	Mica-sand-LBG	M_L_control	-	-	-	120	240	100		0	5/6	0	
		M_L0.12								0.12		0.12	
**Design biopolymer content according to clay content**													
III	Bentonite-sand-LBG/C	B_L/C_control	-	11.1	-	-	22.2	100		0	9/1	-	0
		B_L/C 0.12								0.013			0.12
		B_L/C 0.6								0.067			0.6
		B_L/C 1.2								0.133			1.2
	Vermiculite-sand-LBG/C	V_L/C_control	-	-	50	-	100	100		0	2/1	-	0
		V_L/C 0.12								0.06			0.12
		V_L/C 0.6								0.3			0.6
		V_L/C 1.2								0.6			1.2
**Mica-sand-LBG group supplementary experiments**													
IV	Mica-sand-LBG/E	M_L0.12	-	-	-	100	200	100		0.12	1/1	0.12	0.12
		M_L0.6								0.6		0.6	0.6
		M_L1.2								1.2		1.2	1.2

Table S3.2 The Constant Shear Stress Applied for Creep Tests

Group	Applied constant shear stress for creep tests
Kaolinite-sand-SA	3 Pa
Kaolinite-sand-LBG	10 Pa
Bentonite-sand-SA	50 Pa
Bentonite-sand-LBG	30 Pa
Vermiculite-sand-SA	30 Pa
Vermiculite-sand-LBG	30 Pa
Mica-sand-SA	1 Pa
Mica-sand-LBG	3 Pa

Table S3.3 Mixture Proportions for Mesoscale Printing

Series	Mix ID	Natural Earth (g)	Kaolinite (g)	Bentonite (g)	Vermiculite (g)	Mica (g)	Sand (g) (Ottawa F-75)	Water (g)	SA (g)	LBG (g)	Water/Solid ratio
I	EE_SA_Control	-	21.51	4.36	5.45	23.15	45.53	60	-	-	0.6
	EE_SA 0.12								0.072		
	EE_SA 0.6								0.36		
	EE_SA 1.2								0.72		
	EE_L_Control	-	21.51	4.36	5.45	23.15	45.53	80	-	-	0.8
	EE_L 0.12									0.096	
	EE_L 0.6									0.48	
	EE_L 1.2									0.96	
II	EE_56%	-	21.51	4.36	5.45	23.15	45.53	80	-	-	0.8
	EE_59%							70			0.7
	EE_62%							60			0.6
	EE_67%							50			0.5
	EE_71%							40			0.4
	EE_75%							33.3			0.33
	EE_79%							26.5			0.27
III	NE_67%	100	-	-	-	-	-	50	-	-	0.5
	NE_71%	100						40	-		0.4
	NE_75%	100						33.3	-		0.33
	NE_79%	100						26.5	-		0.27
	NE_SA0.12_67%	100						50	0.12		0.5
	NE_SA0.12_71%	100						40	0.12		0.4
	NE_SA0.12_75%	100						33.3	0.12		0.33
	NE_SA0.12_79%	100						26.5	0.12		0.27
