# Supplementary material for: Bio-inspired 3D-printed earthen materials and structures
Source: Nat Commun. 2026 Apr 18;17:5380. doi: 10.1038/s41467-026-71885-z (PMC13275924; doi:10.1038/s41467-026-71885-z)
Supplement: Supplementary file 2 — Transparent Peer Review file [file 41467_2026_71885_MOESM2_ESM.pdf]

nature portfolio

Peer Review File

Bio-inspired 3D-printed Earthen Materials and Structures

Corresponding Author: Dr Samuel Armistead

This file contains all reviewer reports in order by version, followed by all author rebuttals in order by version.

Version 0:

Reviewer comments:

Reviewer #1

(Remarks to the Author)
This study is much needed in the field of bio-based materials development and manufacturing. An analysis of sand, clay, and biopolymer within the context of bio-stabilized earthen structures offers critical insight into the distinct roles and interactions of each component and enhances the understanding of their collective behavior in biomaterial systems. Printability and physicochemistry results are very relevant, however, the claims of scale-up towards structural applications are lacking detail and data.

Here are needed changes:

— Figure 1 depicts micro, meso, and macro considerations of the research. However the diagrams, specifically in part 1 “Nature’s Approach” and part 2 “A multiscale biomimetic approach” are rather vague in representing materials and interactions. Authors can consider utilizing their imaging results, and physiochemical data in demonstrating “optimal interactions” and “optimal mixtures”

— Rheological analyses of wet blends are relevant for understanding printability. However, mechanical characterization of dry constructs would ultimately reveal this study’s value in scaling up. Mechanical characterization data is needed in compression and bending capacity to elucidate earthen materials’ and binders’ contributions to loading capacity, beyond printability. Following tests are recommended:
— Compressive and bending tests of investigated blends
— Compressive and bending tests of 3D printed constructs

— In the “Printability” section, authors don’t describe the printing set up including nozzle size, pressure, and prototype geometry. However, several print parameters are noted later under the “Biopolymer Effects on Large Scale 3D-Printing” section. The inconsistency in describing 3D printing conditions and parameters across all tests should be addressed by either moving all print set up and parameter descriptions to the methods section, or clearly stating them for each of the aforementioned sections.

— Figure 3 lacks to depict the “goal” geometry (ie the toolpath) and lacks to include scale bars.

— Construction and architecture scale applications are mentioned throughout the text, however, scale up of methods is not discussed since some protocols use small and expensive equipment. How will this research scale into 3d-printed large structures?

— Between lines 278–280, the authors assert that bio-stabilized earthen materials are suitable for use in both pressed monolithic walls and robotically formed bricks. While earthen structures are vaguely alluded to in lines 34-50, specific earthen construction techniques including rammed and robotically formed architectures are not properly introduced or discussed in the manuscript. If the authors intend to claim broad applicability of bio-stabilized earth across diverse construction methods, it is essential that they provide a contextual analysis of current practices in these techniques to substantiate their claim.

— Crucially, although printability and 3D printed structures are central to the research, the paper lacks a review of the state of the art in 3D printed earthen constructs. Incorporating analysis, combined with biomimetic vision for earth bio-stabilization would enhance the depth and contribution of the paper.

— Wet to Dry internal structure analysis via imaging of materials distribution in printed constructs would also be very relevant to this research to determine homogenous distribution of minerals and binders in the extrusion results to be able to claim proper blend design.

— To substantiate the possibility of scaling up, authors should critically consider and address how humidity, temperature, UV radiation and other environmental factors would impact bio-stabilized earthen architectures within architectural and construction applications.

— Claims of methodology for scalability might need to be revisited per suggestions above and perhaps downgraded. Claims could hint at a vision towards scalable construction since scalable logistics are not explored in sufficient detail to be claimed as a result of the research.

Reviewer #2

(Remarks to the Author)
This paper presents a systematic investigation on printing earthen materials by adding biopolymers into printing paste. The combination of mineral-biopolymers, the rheological property of paste and the printed structures were discussed in this paper. However, the addition of polymer in paste is not a novel method in direct ink writing (DIW) printing process (https://doi.org/10.1111/j.1551-2916.2005.00646.x, https://doi.org/10.1038/s41598-017-14236-9, https://doi.org/10.1002/1521-4095(20020916)14:18<1279::AID-ADMA1279>3.0.CO;2-A and so on), authors need to further clarify the novelty of this paper. What's more, authors mentioned the multiscale biomimetic approach, but the optimized effect (like enhancement in mechanical properties) was not shown in the paper, this part maybe need to be supplemented. Although authors made considerable efforts, I think this paper is not suitable to be published now.

Reviewer #3

(Remarks to the Author)
Armistead and coworkers reported the 3d printing of composites, consisted of biopolymers and earthen minerals, utilizing alginate-based biopolymer as stabilizer. They have conducted extensive experiments to reveal the key role of biopolymers in improving the stabilizing those minerals particles, as well as those 3d printing validation. However, using polymers to stabilize the inorganic particles, as well as 3d printing of such nano-/micro-composites has been widely reported, this is not something new at all. Additionally, the authors claimed the biomimetic hierarchical structures, as inspired by those nature materials/structures, However, the complexity and hierarchy 3dp structures are far away from those nature prototypes. Therefore, I can not recommend the publication in nature communications.

Version 1:

Reviewer comments:

Reviewer #1

(Remarks to the Author)
Many thanks for implementing changes.
It is still unclear if the structural claims and construction field mentions are appropriate for the given results and if this article's its scientific contribution is a fundamental science one.

In more detail:
- Size of constructs and tectonics demonstrated by the technique do not show scale up ability of the blends to architectural structures as mentioned.
- It is unclear if compression strength obtained can support scale up to architectural structures depicted in figures.
- Comparison table to raw/fired/bio stabilized printed clays compression data in MPa extracted from literature would help.
- Perhaps focus and claims of the article should be solely on a multiscale framework for systematically investigating and optimizing the use of biopolymer stabilizers in clay.

That being said, the fundamental science developed might not be of enough novelty to be published in this journal.

Reviewer #2

(Remarks to the Author)
The revised version of this manuscript is improved and emphasizes that the novelty of this work is focused on the printing process of earthen systems comprised of clays and sands. However, the introduction of biopolymers into earthen systems is not a novel approach (doi.org/10.1016/j.matt.2023.08.023). This work may not be suitable for publication in the prestigious comprehensive journal Nature Communications. A more specialized journal may be a suitable choice.

Reviewer #4

(Remarks to the Author)
The manuscript submitted by Samuel J. Armistead, entitled “Bio-inspired 3D-printed Earthen Materials and Structures”, has been submitted to Springer Nature. The paper addresses the use of biopolymers to enhance the performance of natural earthen materials for large-scale 3D printing technologies in construction. The targeted properties include processing-related properties (notably rheological behavior), early-age properties (such as drying shrinkage), and long-term mechanical properties (e.g., compressive strength).

This topic is of significant interest, as it aims to replace cement-based materials and chemical admixtures—whose environmental impacts are well documented—with materials derived from natural sources. These natural materials include excavated soils and biopolymers produced through processes that ensure renewability. At a time when the construction industry is actively seeking to reduce its environmental footprint, such solutions offer highly promising perspectives.

Although the manuscript focuses on the use of biopolymer–earthen matrices for 3D printing, the results extend beyond this emerging construction technology. Indeed, the proposed solutions could also be applied to improve the performance of traditional earthen construction techniques such as rammed earth, adobe, or compressed earth blocks, notably by reducing their sensitivity to water and/or enhancing their mechanical properties.

The originality of the presented results lies not only in the materials employed, but also in the experimental and analytical techniques used to investigate and explain the effects of biopolymers. These explanations are grounded in physico-chemical interactions between biopolymers and clays, as well as between biopolymers and sand—typically considered inert, an assumption that is questioned by the authors. This is not a trivial task, given the diversity of clay minerals and the fact that natural soils generally contain multiple clay types in varying proportions.

The manuscript follows a methodology previously introduced by the authors in a recently published review article. Here, this methodology is extended to a broader experimental framework aimed at achieving a deeper understanding of the aforementioned interactions. For instance, the authors investigate different biopolymers and clay types, as well as various sand–clay mixtures, including one mixture designed to reproduce a natural earth, which is also studied. The authors adopt a multiscale approach, combining investigations at the microscopic scale to elucidate mechanisms, mesoscopic-scale experiments (small-scale 3D printing) to assess material behavior, and macroscopic-scale analyses to evaluate printability and constructability (for both natural earth and engineered earth).

The results presented are highly interesting and are supported by a clearly articulated scientific approach, employing methods that allow for a fine-grained understanding of the underlying phenomena. The manuscript goes beyond qualitative explanations or purely technical observations, which are unfortunately still common in the literature on this topic. In this regard, the authors provide the 3D printing research community with a reproducible experimental framework that helps rationalize knowledge-driven research in this field. For all these reasons, I believe that the manuscript merits publication. The revisions made after the first round of review have already improved the manuscript and clarified several important points.

That said, several questions remain regarding certain methodological choices, results, and claims made by the authors:

- Thermogravimetric analysis (TGA): The authors report mass loss measurements for individual components as well as for their mixtures. Would it not be possible to predict a theoretical mass loss in the absence of interactions, based on the mass losses of the isolated materials and their proportions in the mixtures? Such an approach would help demonstrate that the observed differences are indeed due to interactions rather than simple dilution effects.

- Materials: “The natural earth used was a byproduct of excavation operations sourced from a granite quarry in Golden, Colorado (USA).” Is this material truly a natural earth, or does it rather correspond to quarry wash fines? These materials differ substantially in terms of composition, particle size distribution, and consequently mechanical and rheological behavior.

- Particle size distribution: Several methods are used to characterize the particle size distributions of the materials. These methods differ from those commonly employed in soil mechanics, where wet sieving and sedimentation techniques are typically used for clay-rich soils. Here, laser diffraction and image analysis from microscopy are employed. Could the authors clarify why standard soil characterization methods were not used?

- Material preparation: The preparation protocol for clay-containing materials involves high-shear mixing, sonication, and other intensive procedures. This protocol is highly elaborate and appears difficult to implement at the scale required for large-scale 3D printing. What, then, is the representativeness of the obtained results with respect to large-scale material preparation? (For the specific purpose of studying biopolymer–clay interactions, the protocol itself is fully appropriate.)

- Biopolymer retention after washing: “The suspension was then centrifuged at 4000 rpm for 10 minutes, and the supernatant was carefully removed…” After this preparation and repeated removal of the supernatant, is it still possible to determine the actual composition of the resulting material? Do biopolymers remain present in the final preparation, and if so, what is their final mass fraction? Without knowing the exact composition of the mixture, how can robust conclusions be drawn?

- Rheological tests: The authors indicate that the water content is adjusted to allow testing with the rheometer used, but they do not specify the criterion used for this adjustment. What is this criterion, and how can the rheological results be meaningfully compared across different formulations under these conditions?

- Engineered earth formulation: “Since chlorite clay, accounting for 9% of the natural earth composition (Extended Data Fig. 10f), was outside of the minerals explored for this study, it was replaced with 4.5% mica and 4.5% kaolinite…” How can one be confident that this substitution leads to a mechanical and rheological behavior comparable to that of the natural earth?

- Time-dependent behavior: Is a difference in hardening or setting rate observed between the different formulations? How do the rheological properties evolve with time and drying duration? Have the authors investigated the thixotropic behavior of the materials?

Version 2:

Reviewer comments:

Reviewer #4

(Remarks to the Author)
The authors have adequately addressed the various questions and comments raised by the reviewers, and in its current form, the understanding and justification of the study are much clearer. The manuscript can therefore be accepted for publication.

Open Access This Peer Review File is licensed under a Creative Commons Attribution 4.0 International License, which permits use, sharing, adaptation, distribution and reproduction in any medium or format, as long as you give appropriate credit to the original author(s) and the source, provide a link to the Creative Commons license, and indicate if changes were made.
In cases where reviewers are anonymous, credit should be given to 'Anonymous Referee' and the source.
The images or other third party material in this Peer Review File are included in the article’s Creative Commons license, unless indicated otherwise in a credit line to the material. If material is not included in the article’s Creative Commons license and your intended use is not permitted by statutory regulation or exceeds the permitted use, you will need to obtain permission directly from the copyright holder.
To view a copy of this license, visit https://creativecommons.org/licenses/by/4.0/

31st October 2025

Authors' Response to Reviewer Comments

I would like to extend my thanks to the three expert reviewers for their help to improve the manuscript. We hope you share our view that the manuscript is now at a standard for publication and will make an impactful additional to *Nature Communications*. We have addressed all comments point by point in the following document. Our responses are shown in blue text, with line number referring to the 'Armistead et al. (Manuscript - Clean)' version. A tracked-changes version is also provided for completeness.

Reviewer #1 (Remarks to the Author):

This study is much needed in the field of bio-based materials development and manufacturing. An analysis of sand, clay, and biopolymer within the context of bio-stabilized earthen structures offers critical insight into the distinct roles and interactions of each component and enhances the understanding of their collective behavior in biomaterial systems. Printability and physicochemistry results are very relevant, however, the claims of scale-up towards structural applications are lacking detail and data.

The authors very much appreciate the reviewer's supportive words. We hope to have addressed the outstanding comments through the following minor manuscript alterations.

Here are needed changes:

— Figure 1 depicts micro, meso, and macro considerations of the research. However the diagrams, specifically in part 1 "Nature's Approach" and part 2 "A multiscale biomimetic approach" are rather vague in representing materials and interactions. Authors can consider utilizing their imaging results, and physiochemical data in demonstrating "optimal interactions" and "optimal mixtures"

The authors appreciate the reviewer's suggestion and have made adjustments to Figure 1 as recommended.

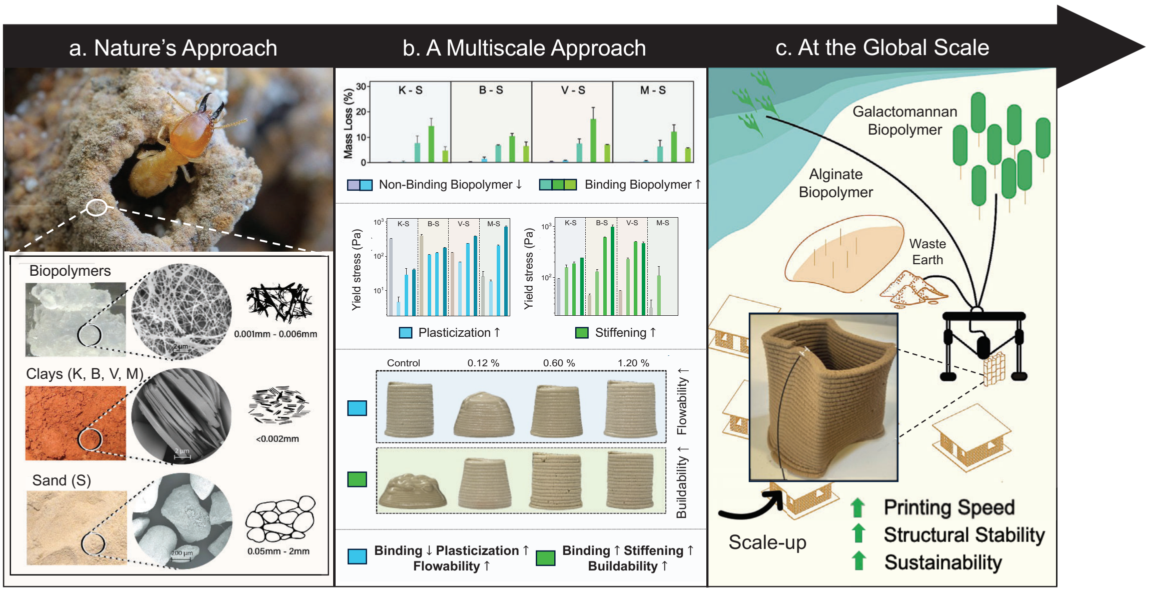

— Rheological analyses of wet blends are relevant for understanding printability. However, mechanical characterization of dry constructs would ultimately reveal this study's value in scaling up. Mechanical characterization data is needed in compression and bending capacity to elucidate earthen materials' and binders' contributions to loading capacity, beyond printability. Following tests are recommended:

— Compressive and bending tests of investigated blends
— Compressive and bending tests of 3D printed constructs

The authors appreciate the valuable feedback provided by the editor and reviewers. We were perhaps not clear in our original submission that the primary property of interest in our study is printability (versus compressive strength). Nonetheless, to address the concerns raised by the Editors and reviewers regarding mechanical property improvement, we have characterized and reported shrinkage and compressive strength measurements in our revised submission. Importantly, we now show that the addition of sodium alginate (SA) improves the drying shrinkage and compressive strength of 3D-printed earth:

L266-272

To validate SA's effects in natural systems, small-scale printing experiments were conducted using natural earth with 0.12% SA at varying earth content. Consistent with engineered earth, SA improved flowability and enabled printing at significantly higher earth contents (Fig. 3k). This formulation resulted in a 75% reduction in drying shrinkage (from 10.31% to 2.61%) and a 25% improvement in compressive strength compared to control prints (Extended Data Fig. 11). Thus, SA 0.12 % and an earth-to-water ratio of 3:1 was used as the starting point for the macroscale investigation (Fig. 4).

L809-816

Extended Data Fig. 11 Syringe-Printed Natural Earth Shrinkage & Compressive Strength. Linear shrinkage of printed samples measured via initial height (25.4mm) against final height of sample following curing. The addition of SA 0.12% enabled an increased earth:water ratio from 2:1 to 3:1 (indicated in parenthesis on figure) which facilitated a significant reduction in shrinkage versus the control from 10.31% to 2.61% and a statistically significant 25% increase in mean compressive strength increase from 1.24 MPa to 1.56 MPa (Welch's t-test, $p = 0.011$). The addition of LBG 0.6% and 1.2% resulted in a non-extrudable mixture, therefore no shrinkage or compressive strength could be measured.

The manuscript has also been carefully reviewed, and any claims associated with widespread structural use have been moved to a discussion of future potential impact:

L315-324

The framework can be expanded to address other material properties, such as density, compressive strength, thermal conductivity, and moisture resistance, for a range of environmental exposure conditions (e.g., humidity, temperature, UV radiation). Enhancing these performance characteristics is essential for the practical implementation of biopolymer-stabilized earth as a widely utilized structural and/or insulation construction material. In addition to 3D-printed elements, the framework can also be employed to optimize

the design and engineering of other earth-based construction methods, including rammed earth walls and compressed earth blocks. By integrating these advancements, researchers, engineers, architects, and builders can develop sustainable solutions that revolutionize construction by using locally sourced earthen materials in place of other resource- and carbon-intensive alternatives.

— In the "Printability" section, authors don't describe the printing set up including nozzle size, pressure, and prototype geometry. However, several print parameters are noted later under the "Biopolymer Effects on Large Scale 3D-Printing" section. The inconsistency in describing 3D printing conditions and parameters across all tests should be addressed by either moving all print set up and parameter descriptions to the methods section, or clearly stating them for each of the aforementioned sections.

The authors thank the reviewer for their comment. The printing parameters for the printability section have been included:

L659-663

Small-Scale Printing. To evaluate the 3D-printability of the prepared mixtures (Supplementary Methods 1.2), cylindrical samples (25.4 mm in diameter and height) were printed using a custom-designed syringe gantry printer, as detailed in reference58 (Extended Data Fig. 10f). This printer was equipped with a 60 ml syringe and a nozzle with a diameter of 2.4 mm. The samples were extruded at a constant rate of 40 mm/s.

The 'Biopolymer Effects on Macroscale 3D-Printing' section discussed the printing parameters (e.g., printing speed, overhang angle, wall thickness) that were optimized with the addition of SA, whereas the other parameters that remained constant were only specified in the methods section.

— Figure 3 lacks to depict the "goal" geometry (ie the toolpath) and lacks to include scale bars.

The authors thank the reviewer for noticing this omission, the scale bar has now been included Figure 3 and the toolpath geometry included in Figure 10j in the extended data.

— Construction and architecture scale applications are mentioned throughout the text, however, scale up of methods is not discussed since some protocols use small and expensive equipment. How will this research scale into 3d-printed large structures?

The authors very much appreciate the suggestion made by the reviewer. We have moved scalability claims to the future outlook section.

L325-332

Furthermore, the multiscale framework provides a foundation for integrating cutting-edge technologies such as synthetic biology (e.g., engineered bio-stabilizers), digital processing (e.g., in-situ imaging and analysis), and artificial intelligence (e.g., predictive performance modeling). This integration will enable the real-time analysis and optimization of large volumes of waste earth with tailored biopolymer stabilizers, an essential step toward the building-scale implementation in insulation, non-loadbearing, and load-bearing

wall assemblies. When further coupled with supply chain, technoeconomic, and environmental analyses, these innovations collectively pave the way for the widespread adoption of bio-inspired, 3D-printed earthen materials and structures.

— Between lines 278–280, the authors assert that bio-stabilized earthen materials are suitable for use in both pressed monolithic walls and robotically formed bricks. While earthen structures are vaguely alluded to in lines 34-50, specific earthen construction techniques including rammed and robotically formed architectures are not properly introduced or discussed in the manuscript. If the authors intend to claim broad applicability of bio-stabilized earth across diverse construction methods, it is essential that they provide a contextual analysis of current practices in these techniques to substantiate their claim.

The authors appreciate and agree with the reviewer's comments. Additional context regarding the application of biopolymer stabilizers in other earthen construction methods has been included in state of the art in the revised manuscript.

L55-66

Biopolymer stabilizers are being utilized worldwide by researchers to transform earthen materials into high-performance, sustainable construction materials[6]. In a recent study, Xu et al.[12] demonstrated that mixtures of quaternized chitosan and alginate polysaccharides could stabilize diverse aggregates, including sand, slag, and crushed brick, and could achieve compressive and flexural strengths up to 17 MPa and 14 MPa, respectively. Kulshreshtha et al.[13] created a "CoRncrete" by mixing corn starch with sand, achieving strengths up to 26 MPa. Christ et al.[14] discovered that the addition of gelatin biopolymers to sand generated compressive strengths of up to 60 MPa and revealed a correlation between adhesive strength and bulk compressive strength performance. While these studies highlight the potential mechanical property enhancements achievable by adding biopolymer stabilizers to earth, they do not necessarily determine or report the underlying chemical and biological mechanisms that underpin the stabilization and property enhancements.

Alternative wording has been used to emphasize that these methods are part of the future outlook for the manuscript's impact.

L319-324

In addition to 3D-printed elements, the framework can also be employed to optimize the design and engineering of other earth-based construction methods, including rammed earth walls and compressed earth blocks. By integrating these advancements, researchers, engineers, architects, and builders can develop sustainable solutions that revolutionize construction by using locally sourced earthen materials in place of other resource- and carbon-intensive alternatives.

— Crucially, although printability and 3D printed structures are central to the research, the paper lacks a review of the state of the art in 3D printed earthen constructs. Incorporating analysis, combined with biomimetic vision for earth bio-stabilization would enhance the depth and contribution of the paper.

The authors appreciate the reviewer's comment and agree that further context of the state-of-the-art and the key step-changes provided by the manuscript would improve the manuscript. Within the new state-of-the-art section, key references have been included and incorporated into the text.

L54-88

State of the Art

Biopolymer stabilizers are being utilized worldwide by researchers to transform earthen materials into high-performance, sustainable construction materials[6]. In a recent study, Xu et al.[12] demonstrated that mixtures of quaternized chitosan and alginate polysaccharides could stabilize diverse aggregates, including sand, slag, and crushed brick, and could achieve compressive and flexural strengths up to 17 MPa and 14 MPa, respectively. Kulshreshtha et al.[13] created a "CoRncrete" by mixing corn starch with sand, achieving strengths up to 26 MPa. Christ et al.[14] discovered that the addition of gelatin biopolymers to sand generated compressive strengths of up to 60 MPa and revealed a correlation between adhesive strength and bulk compressive strength performance. While these studies highlight the potential mechanical property enhancements achievable by adding biopolymer stabilizers to earth, they do not necessarily determine or report the underlying chemical and biological mechanisms that underpin the stabilization and property enhancements.

Armistead et al.[15] pioneered a new characterization protocol for earthen materials that elucidates the underlying chemical and biological interactions between minerals and biopolymers and links those interactions to bulk material properties. Using the protocol, the authors discovered that increased binding affinity between galactomannan biopolymers and iron oxide (Fe_2O_3) correlated directly with increases in compressive strength[16]. Mikofsky et al.[17] employed the protocol to discover that biopolymer binding affinity was predominantly inversely correlated with compressive strength of bentonite and kaolinite clays. In Maierdan et al.[18] the protocol was applied for the first time to study the influence of alginate on the 3D printing properties of kaolinite rheological and small-scale extrusion experiments revealed that alginate-driven electrostatic repulsions improved the printing performance of kaolinite-based earthen materials. While these advances are notable, the findings to-date have been limited in (1) universality[15-18] (i.e., applicability to multiple clays and biopolymer systems), (2) complexity[19] (i.e., applicability to engineered or natural clay-sand systems), and (3) scale[20–23] (i.e., applicability to macroscale 3D printing).

Collectively, the current state of the art highlights both the promise of biopolymer-stabilized 3D-printed earthen materials and the persistent technological challenges that limit their widespread application. This dichotomy underscores the urgent need for an experimental framework that interrogates molecular interactions, mixture formulations, and printing parameters, thus establishing a pathway from fundamental chemical building blocks to architecturally relevant structures. In this study, we address this gap by developing and deploying a multiscale framework for systematically investigating and optimizing the use of biopolymer stabilizers to address key challenges associated with 3D-printed earthen materials and structures.

— Wet to Dry internal structure analysis via imaging of materials distribution in printed constructs would also be very relevant to this research to determine homogenous distribution of minerals and binders in the extrusion results to be able to claim proper blend design.

The authors appreciate the suggestion, which has been alluded to in the outlook section:

L325-332

Furthermore, the multiscale framework provides a foundation for integrating cutting-edge technologies such as synthetic biology (e.g., engineered bio-stabilizers), digital processing (e.g., in-situ imaging and analysis), and artificial intelligence (e.g., predictive performance modeling). This integration will enable the real-time analysis and optimization of large volumes of waste earth with tailored biopolymer stabilizers, an essential step toward the building-scale implementation in insulation, non-loadbearing, and load-bearing wall assemblies. When further coupled with supply chain, technoeconomic, and environmental analyses, these innovations collectively pave the way for the widespread adoption of bio-inspired, 3D-printed earthen materials and structures.

— To substantiate the possibility of scaling up, authors should critically consider and address how humidity, temperature, UV radiation and other environmental factors would impact bio-stabilized earthen architectures within architectural and construction applications.

The authors very much appreciate this suggestion from the reviewer and have included the suggestions within the future impact section of the manuscript:

L315-324

The framework can be expanded to address other material properties, such as density, compressive strength, thermal conductivity, and moisture resistance, for a range of environmental exposure conditions (e.g., humidity, temperature, UV radiation). Enhancing these performance characteristics is essential for the practical implementation of biopolymer-stabilized earth as a widely utilized structural and/or insulation construction material.

— Claims of methodology for scalability might need to be revisited per suggestions above and perhaps downgraded. Claims could hint at a vision towards scalable construction since scalable logistics are not explored in sufficient detail to be claimed as a result of the research.

The authors very much appreciate the suggestion made by the reviewer. We have moved scalability claims to the future outlook section.

L325-332

Furthermore, the multiscale framework provides a foundation for integrating cutting-edge technologies such as synthetic biology (e.g., engineered bio-stabilizers), digital processing (e.g., in-situ imaging and analysis), and artificial intelligence (e.g., predictive performance modeling). This integration will enable

the real-time analysis and optimization of large volumes of waste earth with tailored biopolymer stabilizers, an essential step toward the building-scale implementation in insulation, non-loadbearing, and load-bearing wall assemblies. When further coupled with supply chain, technoeconomic, and environmental analyses, these innovations collectively pave the way for the widespread adoption of bio-inspired, 3D-printed earthen materials and structures.

Reviewer #2 (Remarks to the Author):

This paper presents a systematic investigation on printing earthen materials by adding biopolymers into printing paste. The combination of mineral-biopolymers, the rheological property of paste and the printed structures were discussed in this paper.

We thank the reviewer for taking the time to review the manuscript.

However, the addition of polymer in paste is not a novel method in direct ink writing (DIW) printing process (https://doi.org/10.1111/j.1551-2916.2005.00646.x, https://doi.org/10.1038/s41598-017-14236-9, https://doi.org/10.1002/1521-4095(20020916)14:18<1279::AID-ADMA1279>3.0.CO;2-A and so on), authors need to further clarify the novelty of this paper.

We appreciate the opportunity to clarify the novelty of this paper.

In DIW, polymers are typically blended with homogeneous, inert ceramic or polymeric fillers, producing predictable and well-characterized rheological effects for small-scale 3D-printing. In contrast, our work focuses on biopolymer-stabilization of earthen systems comprised of clays and sands, whose interfacial properties and natural origins present unique physical and chemical challenges, which we interrogate to enable 3D-printing at the macroscale.

In this manuscript, we develop and deploy a rigorous experimental framework to elucidate how microscale biopolymer-earth interactions dictate material behaviour at the meso- and macroscales. This unifying framework links interfacial phenomena to the performance of macroscale 3D printing with natural, heterogeneous earthen materials.

We have included a state-of-the-art section in the introduction to emphasise the novelty of the manuscript:

L54-88

State of the Art

Biopolymer stabilizers are being utilized worldwide by researchers to transform earthen materials into high-performance, sustainable construction materials[6]. In a recent study, Xu et al.[12] demonstrated that mixtures of quaternized chitosan and alginate polysaccharides could stabilize diverse aggregates, including sand, slag, and crushed brick, and could achieve compressive and flexural strengths up to 17 MPa and 14 MPa, respectively. Kulshreshtha et al.[13] created a "CoRncrete" by mixing corn starch with sand, achieving strengths up to 26 MPa. Christ et al.[14] discovered that the addition of gelatin biopolymers to sand generated compressive strengths of up to 60 MPa and revealed a correlation between adhesive strength and bulk compressive strength performance. While these studies highlight the potential mechanical property enhancements achievable by adding biopolymer stabilizers to earth, they do not necessarily determine or report the underlying chemical and biological mechanisms that underpin the stabilization and property enhancements.

Armistead et al.[15] pioneered a new characterization protocol for earthen materials that elucidates the underlying chemical and biological interactions between minerals and biopolymers and links those interactions to bulk material properties. Using the protocol, the authors discovered that increased binding affinity between galactomannan biopolymers and iron oxide (Fe_2O_3) correlated directly with increases in compressive strength[16]. Mikofsky et al.[17] employed the protocol to discover that biopolymer binding affinity was predominantly inversely correlated with compressive strength of bentonite and kaolinite clays. In Maierdan et al.[18] the protocol was applied for the first time to study the influence of alginate on the 3D printing properties of kaolinite rheological and small-scale extrusion experiments revealed that alginate-driven electrostatic repulsions improved the printing performance of kaolinite-based earthen materials. While these advances are notable, the findings to-date have been limited in (1) universality[15-18] (i.e., applicability to multiple clays and biopolymer systems), (2) complexity[19] (i.e., applicability to engineered or natural clay-sand systems), and (3) scale[20–23] (i.e., applicability to macroscale 3D printing).

Collectively, the current state of the art highlights both the promise of biopolymer-stabilized 3D-printed earthen materials and the persistent technological challenges that limit their widespread application. This dichotomy underscores the urgent need for an experimental framework that interrogates molecular interactions, mixture formulations, and printing parameters, thus establishing a pathway from fundamental chemical building blocks to architecturally relevant structures. In this study, we address this gap by developing and deploying a multiscale framework for systematically investigating and optimizing the use of biopolymer stabilizers to address key challenges associated with 3D-printed earthen materials and structures.

What's more, authors mentioned the multiscale biomimetic approach, but the optimized effect (like enhancement in mechanical properties) was not shown in the paper, this part maybe need to be supplemented.

The authors appreciate the valuable feedback provided by the reviewer. We were perhaps not clear in our original submission that the primary property of interest in our study is printability (versus compressive strength). Nonetheless, to address the concerns raised by the Editors and reviewers regarding mechanical property improvement, we have characterized and reported shrinkage and compressive strength measurements in our revised submission. Importantly, we now show that the addition of sodium alginate (SA) improves the drying shrinkage and compressive strength of 3D-printed earth:

L266-272

To validate SA's effects in natural systems, small-scale printing experiments were conducted using natural earth with 0.12% SA at varying earth content. Consistent with engineered earth, SA improved flowability and enabled printing at significantly higher earth contents (Fig. 3k). This formulation resulted in a 75% reduction in drying shrinkage (from 10.31% to 2.61%) and a 25% improvement in compressive strength compared to control prints (Extended Data Fig. 11). Thus, SA 0.12 % and an earth-to-water ratio of 3:1 was used as the starting point for macroscale investigation (Fig. 4).

L809-816

Extended Data Fig. 11 Syringe-Printed Natural Earth Shrinkage & Compressive Strength. Linear shrinkage of printed samples measured via initial height (25.4mm) against final height of sample following curing. The addition of SA 0.12% enabled an increased earth:water ratio from 2:1 to 3:1 (indicated in parenthesis on figure) which facilitated a significant reduction in shrinkage versus the control from 10.31% to 2.61% and a statistically significant 25% increase in mean compressive strength increase from 1.24 MPa to 1.56 MPa (Welch's t-test, $p = 0.011$). The addition of LBG 0.6% and 1.2% resulted in a non-extrudable mixture, therefore no shrinkage or compressive strength could be measured.

The manuscript has also been carefully reviewed, and any claims associated with widespread structural use have been moved to a discussion of future potential impact:

L315-324

The framework can be expanded to address other material properties, such as density, compressive strength, thermal conductivity, and moisture resistance, for a range of environmental exposure conditions (e.g., humidity, temperature, UV radiation). Enhancing these performance characteristics is essential for the practical implementation of biopolymer-stabilized earth as a widely utilized structural and/or insulation construction material. In addition to 3D-printed elements, the framework can also be employed to optimize the design and engineering of other earth-based construction methods, including rammed earth walls and compressed earth blocks. By integrating these advancements, researchers, engineers, architects, and builders can develop sustainable solutions that revolutionize construction by using locally sourced earthen materials in place of other resource- and carbon-intensive alternatives.

Although authors made considerable efforts, I think this paper is not suitable to be published now.

We hope that these edits and clarifications enable the reviewer to appreciate the novelty of our work and to find our manuscript now suitable for publication in Nature Communications.

Reviewer #3 (Remarks to the Author):

Armistead and coworkers reported the 3d printing of composites, consisted of biopolymers and earthen minerals, utilizing alginate-based biopolymer as stabilizer. They have conducted extensive experiments to reveal the key role of biopolymers in improving the stabilizing those minerals particles, as well as those 3d printing validation.

We thank the reviewer for taking the time to review the manuscript.

However, using polymers to stabilize the inorganic particles, as well as 3d printing of such nano-/micro-composites has been widely reported, this is not something new at all.

We appreciate the opportunity to clarify the novelty of this paper.

Polymer-inorganic composites are typically blended with homogeneous, inert ceramic or polymeric fillers, producing predictable and well-characterized rheological effects for small-scale 3D-printing. In contrast, our work focuses on biopolymer-stabilization of earthen systems comprised of clays and sands, whose interfacial properties and natural origins present unique physical and chemical challenges, which we interrogate to enable 3D-printing at the macroscale.

In this manuscript, we develop and deploy a rigorous experimental framework to elucidate how microscale biopolymer-earth interactions dictate material behavior at the meso- and macroscales. This unifying framework links interfacial phenomena to the performance of macroscale 3D printing with natural, heterogeneous earthen materials.

We have included a state-of-the-art section in the introduction to emphasise the novelty of the manuscript:

L54-88

State of the Art

Biopolymer stabilizers are being utilized worldwide by researchers to transform earthen materials into high-performance, sustainable construction materials[6]. In a recent study, Xu et al.[12] demonstrated that mixtures of quaternized chitosan and alginate polysaccharides could stabilize diverse aggregates, including sand, slag, and crushed brick, and could achieve compressive and flexural strengths up to 17 MPa and 14 MPa, respectively. Kulshreshtha et al.[13] created a “CoRncrete” by mixing corn starch with sand, achieving strengths up to 26 MPa. Christ et al.[14] discovered that the addition of gelatin biopolymers to sand generated compressive strengths of up to 60 MPa and revealed a correlation between adhesive strength and bulk compressive strength performance. While these studies highlight the potential mechanical property enhancements achievable by adding biopolymer stabilizers to earth, they do not necessarily determine or report the underlying chemical and biological mechanisms that underpin the stabilization and property enhancements.

Armistead et al.[15] pioneered a new characterization protocol for earthen materials that elucidates the underlying chemical and biological interactions between minerals and biopolymers and links those interactions to bulk material properties. Using the protocol, the authors discovered that increased binding affinity between galactomannan biopolymers and iron oxide (Fe_2O_3) correlated directly with increases in compressive strength[16]. Mikofsky et al.[17] employed the protocol to discover that biopolymer binding affinity was predominantly inversely correlated with compressive strength of bentonite and kaolinite clays. In Maierdan et al.[18] the protocol was applied for the first time to study the influence of alginate on the 3D printing properties of kaolinite rheological and small-scale extrusion experiments revealed that alginate-driven electrostatic repulsions improved the printing performance of kaolinite-based earthen materials. While these advances are notable, the findings to-date have been limited in (1) universality[15-18] (i.e., applicability to multiple clays and biopolymer systems), (2) complexity[19] (i.e., applicability to engineered or natural clay-sand systems), and (3) scale[20–23] (i.e., applicability to macroscale 3D printing).

Collectively, the current state of the art highlights both the promise of biopolymer-stabilized 3D-printed earthen materials and the persistent technological challenges that limit their widespread application. This dichotomy underscores the urgent need for an experimental framework that interrogates molecular interactions, mixture formulations, and printing parameters, thus establishing a pathway from fundamental chemical building blocks to architecturally relevant structures. In this study, we address this gap by developing and deploying a multiscale framework for systematically investigating and optimizing the use of biopolymer stabilizers to address key challenges associated with 3D-printed earthen materials and structures.

Additionally, the authors claimed the biomimetic hierarchical structures, as inspired by those nature materials/structures, However, the complexity and hierarchy 3dp structures are far away from those nature prototypes.

The authors appreciate the suggestion made by the reviewer and agree that the word biomimetic may not be appropriate for this study. Therefore, the authors have changed the title of the manuscript from biomimetic to bio-inspired and adjusted the references to biomimetic throughout the manuscript accordingly.

Therefore, I can not recommend the publication in nature communications.

We hope that these edits and clarifications enable the reviewer to appreciate the novelty of our work and to find our manuscript now suitable for publication in Nature Communications

27th January 2026

Authors' Response to Reviewer Comments

We thank the two reviewers for their continued time and effort in re-reviewing our manuscript, as well as the additional reviewer for their valuable assessment. We hope that our revisions adequately address all comments and that the manuscript is now suitable for publication, making a meaningful contribution to Nature Communications. All comments have been addressed point by point in the following document. Our responses are shown in blue text, with line numbers referring to the Armistead et al. (Manuscript) version. A tracked-changes version is also provided for completeness.

Reviewer #1 (Remarks to the Author):

Many thanks for implementing changes.

It is still unclear if the structural claims and construction field mentions are appropriate for the given results and if this article's scientific contribution is a fundamental science one.

The authors very much appreciate the reviewer's feedback. We hope to have more clearly addressed the outstanding comments through the following responses and minor manuscript alterations.

In more detail:

- Size of constructs and tectonics demonstrated by the technique do not show scale up ability of the blends to architectural structures as mentioned.

We thank the reviewer for this comment and agree that the term architectural structures implies a scale not directly explored in this study. The manuscript has therefore been revised to use the term *architecturally relevant structures*. As illustrated in Fig. 1c, the printed constructs are 3D-printed, brick-scale modular structures (105 × 105 × 105 mm) intended for assembly into architectural structures (i.e., wall assemblies).

L27-28
L88-89
L345-348

- It is unclear if compression strength obtained can support scale up to architectural structures depicted in figures.

The authors thank the reviewer for the comment. In this study, the multiscale framework was used to optimize printing properties rather than mechanical properties. Our future work will undoubtedly extend the approach to other parameters and properties (e.g., compressive strength) critical for the practical implementation of 3D-printed biopolymer-stabilized earth as a widely utilized structural and/or insulative construction material.

To clarify the scope of the work, additional qualifying language has been incorporated through the manuscript (L97: "a key step toward"; L319: "represents a key step toward").

This has been included in future impact:

L321-328:

Future studies can extend this approach to a broader range of processing parameters (i.e., structural build-up, thixotropy) and material properties (i.e., density, mechanical performance, thermal conductivity, and moisture resistance) under relevant environmental exposure conditions (i.e., humidity, temperature, and UV radiation), which are critical for the practical implementation of 3D-printed biopolymer-stabilized earth as a structural and/or insulation construction material. Importantly, the framework can be applied to optimize compressive strength, providing a pathway toward achieving the high strengths previously observed in bio-stabilized earth (up to 60 MPa) within 3D-printed structures.

- Comparison table to raw/fired/bio stabilized printed clays compression data in MPa extracted from literature would help.

The authors thank the reviewer for this comment. To the best of the authors' knowledge, there are limited studies that systematically assess the compressive strength of 3D-printed biopolymer-stabilized earthen materials. This gap in literature, along with our current focus on printing parameters rather than compressive strength, makes direct comparisons to other compressive strength data difficult and tangential to our primary scientific advance. Nevertheless, we highlight the state of the art of high compressive strength earthen materials in our revised manuscript:

L55-67:

Biopolymer stabilizers are being utilized worldwide by researchers to transform earthen materials into high-performance, sustainable construction materials[6]. In a recent study, Xu et al.[12] demonstrated that mixtures of quaternized chitosan and alginate polysaccharides could stabilize diverse aggregates, including sand, slag, and crushed brick, attaining compressive and flexural strengths up to 17 MPa and 14 MPa, respectively. Kulshreshtha et al.[13] created a "CoRncrete" by mixing corn starch with sand, achieving strengths up to 26 MPa. Christ et al.[14] discovered that the addition of gelatin biopolymers to sand generated compressive strengths of up to 60 MPa and revealed a correlation between adhesive strength and bulk compressive strength performance. While these studies report substantial strength gains over unstabilized earth (0.3–7 MPa), with values approaching or in some cases exceeding the minimum strength levels commonly associated with structural masonry (~14 MPa) and concrete (~20 MPa), they do not necessarily determine or report the underlying chemical and biological mechanisms that underpin the stabilization and property enhancements[15–17].

We further highlight that optimizing for compressive strength and other material properties (e.g. thermal conductivity) must be a critical focus of future work:

L321-328:

Future studies can extend this approach to a broader range of processing parameters (i.e., structural build-up, thixotropy) and material properties (i.e., density, mechanical performance, thermal conductivity, and moisture resistance) under relevant environmental exposure conditions (i.e., humidity, temperature, and UV radiation), which are critical for the practical implementation of 3D-printed biopolymer-stabilized earth as a structural and/or insulation construction material. Importantly, the framework can be applied to optimize compressive strength, providing a pathway toward achieving the high strengths previously observed in bio-stabilized earth (up to 60 MPa) within 3D-printed structures.

- Perhaps focus and claims of the article should be solely on a multiscale framework for systematically investigating and optimizing the use of biopolymer stabilizers in clay.

The authors agree that the central contribution of this manuscript is the development and validation of a multiscale framework for systematically investigating and optimizing biopolymer stabilizers in 3D-printed earthen materials and structures. This focus is emphasized throughout the manuscript, while considerations related to broader architectural adoption are discussed in the context of future impacts.

We have emphasised our focus and principal contributions in the introduction:

L54-91:

Biopolymer stabilizers are being utilized worldwide by researchers to transform earthen materials into high-performance, sustainable construction materials[6]. In a recent study, Xu et al.[12] demonstrated that mixtures of quaternized chitosan and alginate polysaccharides could stabilize diverse aggregates, including sand, slag, and crushed brick, attaining compressive and flexural strengths up to 17 MPa and 14 MPa, respectively. Kulshreshtha et al.[13] created a "CoRncrete" by mixing corn starch with sand, achieving strengths up to 26 MPa. Christ et al.[14] discovered that the addition of gelatin biopolymers to sand generated compressive strengths of up to 60 MPa and revealed a correlation between adhesive strength and bulk compressive strength performance. While these studies report substantial strength gains over unstabilized earth (0.3–7 MPa), with values approaching or in some cases exceeding the minimum strength levels commonly associated with structural masonry (~14 MPa) and concrete (~20 MPa), they do not necessarily determine or report the underlying chemical and biological mechanisms that underpin the stabilization and property enhancements[15–17].

Armistead et al.[18] pioneered a new characterization protocol for earthen materials that elucidates the underlying chemical and biological interactions between minerals and biopolymers and links those interactions to bulk material properties. Using the protocol, the authors discovered that increased binding affinity between galactomannan biopolymers and iron oxide (Fe_2O_3) correlated directly with increases in compressive strength[19, 20]. Mikofsky et al.[21] employed the protocol to discover that biopolymer binding affinity was predominantly inversely correlated with compressive strength of bentonite and kaolinite clays. In Maierdan et al.[22] the protocol was applied for the first time to study the influence of alginate on the 3D-printing properties of kaolinite, with rheological and small-scale extrusion experiments revealing that alginate-driven electrostatic repulsions improved the printing performance of kaolinite-based earthen materials. More recently, the protocol was further applied to locust bean gum-kaolinite systems,

demonstrating that biopolymer bridging interactions can enhance underwater extrusion behavior in small-scale experiments[23]. While these advances are notable, the findings to-date have been limited in (1) universality[18-23] (i.e., applicability to multiple clays and biopolymer systems), (2) complexity[24] (i.e., applicability to engineered or natural clay-sand systems), and (3) scale[25–27] (i.e., applicability to macroscale 3D-printing).

Collectively, the current state of the art highlights both the promise of biopolymer-stabilized 3D-printed earthen materials and the persistent technological challenges that limit their widespread application. This dichotomy underscores the urgent need for an experimental framework that interrogates molecular interactions, mixture formulations, and printing parameters, thus establishing a pathway from fundamental chemical building blocks to architecturally relevant structures. In this study, we address this gap by developing and deploying a multiscale framework for systematically investigating and optimizing the use of biopolymer stabilizers to address key challenges associated with 3D-printed earthen materials and structures.

And in the future impact:

L312-333:
By designing and implementing a multiscale optimization framework, the performance of 3D-printed earthen materials and structures has been significantly enhanced, characterized by faster production rates and improved structural stability. This framework has demonstrated its ability to generate foundational insights across multiple scales and disciplines, ultimately converging on an optimal alginate-based biopolymer-stabilizer to improve the 3D-printing properties of diverse and heterogeneous natural earth materials. The framework's ability to quantitatively assess and optimize for the universality, complexity, and scalability of biopolymer-stabilized earth represents a key step toward the production of high-performance, 3D-printed earthen structures at the global scale.

Future studies can extend this approach to a broader range of processing parameters (i.e., structural build-up, thixotropy) and material properties (i.e., density, mechanical performance, thermal conductivity, and moisture resistance) under relevant environmental exposure conditions (i.e., humidity, temperature, and UV radiation), which are critical for the practical implementation of 3D-printed biopolymer-stabilized earth as a structural and/or insulation construction material. Importantly, the framework can be applied to optimize compressive strength, providing a pathway toward achieving the high strengths previously observed in bio-stabilized earth (up to 60 MPa) within 3D-printed structures. In addition to 3D-printed elements, the framework can also be employed to optimize the design and engineering of other earth-based construction methods, including rammed earth walls and compressed earth blocks. By integrating these advancements, researchers, engineers, architects, and builders can develop sustainable solutions that revolutionize construction by using locally sourced earthen materials in place of other resource- and carbon-intensive alternatives.

That being said, the fundamental science developed might not be of enough novelty to be published in this journal.

We appreciate the reviewer's perspective but respectfully disagree with the assessment that the manuscript lacks sufficient novelty for publication in *Nature Communications*. As noted by the reviewer, a central contribution of this work is the introduction of a previously absent, experimentally validated multiscale framework that elucidates and optimizes biopolymer-earth mineral interactions at the microscale and, critically, systematically translates these interactions across length scales. This approach directly addresses longstanding challenges in biopolymer-stabilized 3D-printed earthen materials, namely universality, complexity, and scalability, that have thus far limited both scientific understanding and practical adoption.

Further, by replacing costly, case-specific trial-and-error approaches with a low-cost, rapid, multiscale optimization strategy, this framework represents a paradigm shift in the field. We show that the approach generates new foundational insights across scales, leading to substantial improvements in 3D-printing performance. The framework can now be readily applied to optimize a wide range of material properties, accelerating the widespread global adoption of biopolymer-stabilized, 3D-printed earthen materials.

We believe this combination of conceptual advance, experimental validation, and practical impact aligns well with the aims of *Nature Communications* and will be of broad interest and impact to the journal's readership.

Reviewer #2 (Remarks to the Author):

The revised version of this manuscript is improved and emphasizes that the novelty of this work is focused on the printing process of earthen systems comprised of clays and sands.

We thank the reviewer for their supportive feedback, and hope that through the following comments the novelty of the manuscript is more clearly conveyed.

However, the introduction of biopolymers into earthen systems is not a novel approach (doi.org/10.1016/j.matt.2023.08.023).

We agree with the reviewer on this particular point. However, as we articulate in our manuscript, while the introduction of biopolymer-stabilizers into earthen materials is a growing field, current approaches, including the study referenced by the reviewer (doi.org/10.1016/j.matt.2023.08.023), are largely based on costly trial-and-error investigations or are limited to single biopolymer-earth combinations. In contrast, this study is the first to employ a low-cost, rapid, multiscale framework specifically developed for 3D printing, spanning five biopolymer-stabilizers, five representative clay minerals, and sand, representing approximately 90% of global earth-relevant minerals.

In addition to dramatically increasing experimental throughput and reducing cost, the framework introduces a previously absent, experimentally validated multiscale approach that elucidates and optimizes biopolymer-earth mineral interactions at the microscale and, critically, systematically translates these interactions across length scales. In doing so, it directly addresses longstanding challenges in biopolymer-stabilized 3D-printed earthen materials, namely universality, complexity, and scalability, that have thus far limited both scientific understanding and practical adoption.

The manuscript's contribution also extends beyond framework development. We demonstrate that this multiscale approach yields new foundational insights across scales while simultaneously enabling substantial improvements in printing performance, including increased printing speeds and enhanced structural stability. We believe this combination of experimental validation, conceptual advance, and practical impact aligns well with the aims of *Nature Communications* and will be of broad interest and impact to the journal's readership.

This work may not be suitable for publication in the prestigious comprehensive journal *Nature Communications*. A more specialized journal may be a suitable choice.

We hope that these clarifications address the reviewer's concerns and demonstrate that this manuscript aligns with the aims of *Nature Communications* and will be of broad interest and impact to the journal's readership.

Reviewer #4 (Remarks to the Author):

The manuscript submitted by Samuel J. Armistead, entitled "Bio-inspired 3D-printed Earthen Materials and Structures", has been submitted to Springer Nature. The paper addresses the use of biopolymers to enhance the performance of natural earthen materials for large-scale 3D printing technologies in construction. The targeted properties include processing-related properties (notably rheological behavior), early-age properties (such as drying shrinkage), and long-term mechanical properties (e.g., compressive strength).

This topic is of significant interest, as it aims to replace cement-based materials and chemical admixtures—whose environmental impacts are well documented—with materials derived from natural sources. These natural materials include excavated soils and biopolymers produced through processes that ensure renewability. At a time when the construction industry is actively seeking to reduce its environmental footprint, such solutions offer highly promising perspectives.

Although the manuscript focuses on the use of biopolymer–earthen matrices for 3D printing, the results extend beyond this emerging construction technology. Indeed, the proposed solutions could also be applied to improve the performance of traditional earthen construction techniques such as rammed earth, adobe, or compressed earth blocks, notably by reducing their sensitivity to water and/or enhancing their mechanical properties.

The originality of the presented results lies not only in the materials employed, but also in the experimental and analytical techniques used to investigate and explain the effects of biopolymers. These explanations are grounded in physico-chemical interactions between biopolymers and clays, as well as between biopolymers and sand—typically considered inert, an assumption that is questioned by the authors. This is not a trivial task, given the diversity of clay minerals and the fact that natural soils generally contain multiple clay types in varying proportions.

The manuscript follows a methodology previously introduced by the authors in a recently published review article. Here, this methodology is extended to a broader experimental framework aimed at achieving a deeper understanding of the aforementioned interactions. For instance, the authors investigate different biopolymers and clay types, as well as various sand–clay mixtures, including one mixture designed to reproduce a natural earth, which is also studied. The authors adopt a multiscale approach, combining investigations at the microscopic scale to elucidate mechanisms, mesoscopic-scale experiments (small-scale 3D printing) to assess material behavior, and macroscopic-scale analyses to evaluate printability and constructability (for both natural earth and engineered earth).

The results presented are highly interesting and are supported by a clearly articulated scientific approach, employing methods that allow for a fine-grained understanding of the underlying phenomena. The manuscript goes beyond qualitative explanations or purely technical observations, which are unfortunately still common in the literature on this topic. In this regard, the authors provide the 3D printing research community with a reproducible experimental framework that helps rationalize knowledge-driven research in this field. For all these reasons, I believe that the manuscript merits publication. The revisions made after the first round of review have already improved the manuscript and clarified several important points.

We thank the reviewer for their supportive feedback and hope that the following responses address the remaining questions regarding our methodological choices, results, and claims, and clarify that the manuscript is now suitable for publication in *Nature Communications*.

That said, several questions remain regarding certain methodological choices, results, and claims made by the authors:

- Thermogravimetric analysis (TGA): The authors report mass loss measurements for individual components as well as for their mixtures. Would it not be possible to predict a theoretical mass loss in the absence of interactions, based on the mass losses of the isolated materials and their proportions in the mixtures? Such an approach would help demonstrate that the observed differences are indeed due to interactions rather than simple dilution effects.

The authors appreciate the reviewer's comment. In the present system, any biopolymers that do not participate in interactions are removed during the washing steps prior to TGA analysis. As a result, the remaining material and its associated mass loss originate from interacting components rather than from a simple physical mixture, rendering dilution effects negligible.

This approach does not permit the construction of a theoretical "non-interacting" mass-loss baseline from isolated components, as non-interacting material is intentionally eliminated prior to analysis. For completeness, the TGA data for each individual mineral has been normalized according to their proportion in the final clay-sand mixture, which is provided below (Figure 1). These data reinforce and reflect the trends observed in the non-adjusted figures and discussed in the manuscript.

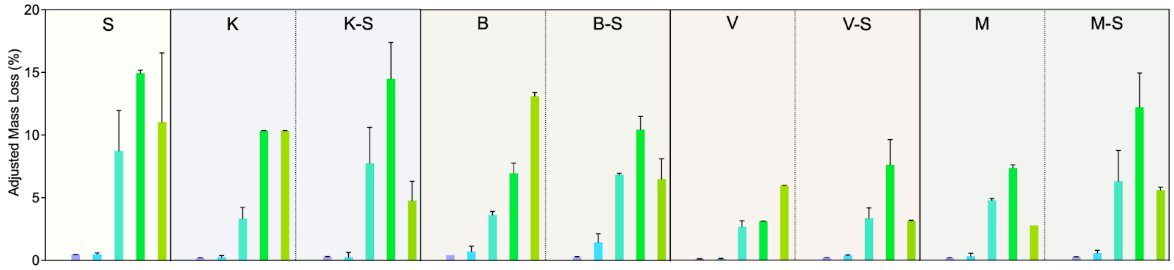

Figure 1. Adjusted theoretical mass loss (200-400 °C) of biopolymer-mineral binding characterization samples based on mineral proportions in clay-sand mixtures.

- Materials: "The natural earth used was a byproduct of excavation operations sourced from a granite quarry in Golden, Colorado (USA)." Is this material truly a natural earth, or does it rather correspond to quarry wash fines? These materials differ substantially in terms of composition, particle size distribution, and consequently mechanical and rheological behavior.

The authors thank the reviewer for the comment. The earth material used in this study was excavated subsoil obtained as a by-product of earth-moving operations at a granite quarry, not quarry wash fines produced through aggregate washing. The materials composition and particle size distribution are consistent with natural subsoil.

- Particle size distribution: Several methods are used to characterize the particle size distributions of the materials. These methods differ from those commonly employed in soil mechanics, where wet sieving and sedimentation techniques are typically used for clay-rich soils. Here, laser diffraction and image analysis from microscopy are employed. Could the authors clarify why standard soil characterization methods were not used?

The authors thank the reviewer for the comment. Standard soil mechanics methods such as wet sieving and sedimentation were not employed because the biopolymer-mineral binding characterization samples were available only in limited quantities, rendering these techniques impractical. Laser diffraction was therefore selected for both the characterization of these binding samples and general soil particle size analysis, as it is a widely used and well-established technique capable of analyzing particle sizes from approximately 0.01 µm to 3500 µm, while offering rapid data acquisition, high reproducibility, and minimal sample requirements.

- Material preparation: The preparation protocol for clay-containing materials involves high-shear mixing, sonication, and other intensive procedures. This protocol is highly elaborate and appears difficult to implement at the scale required for large-scale 3D printing. What, then, is the representativeness of the obtained results with respect to large-scale material preparation? (For the specific purpose of studying biopolymer–clay interactions, the protocol itself is fully appropriate.)

The authors thank the reviewer for the comment. The use of sonication is limited to the microscale and is intended solely to investigate fundamental biopolymer-clay interactions, which, as noted by the reviewer, is appropriate. At the meso- and macroscales, biopolymers were incorporated into earthen materials using conventional physical mixing methods (hand mixing or planetary mixing). These approaches are representative of how biopolymers are typically incorporated into earthen materials in practice.

For clarity, the relevant preparation procedures previously included in the Supplementary Information have been moved to the main manuscript under *Microscale Methods* (*Biopolymer Solution Preparation*) and *Mesoscale Methods (Mix Preparation)*.

- Biopolymer retention after washing: "The suspension was then centrifuged at 4000 rpm for 10 minutes, and the supernatant was carefully removed…" After this preparation and repeated removal of the supernatant, is it still possible to determine the actual composition of the resulting material? Do biopolymers remain present in the final preparation, and if so, what is their final mass fraction? Without knowing the exact composition of the mixture, how can robust conclusions be drawn?

The authors thank the reviewer for the comment. The washing steps are used exclusively within the microscale methods and serve to remove any transient or non-bound biopolymer that could artificially elevate the measured binding affinities. The mass fraction is determined through TGA analysis of the resulting pellet, which provides the exact composition of the mixture, and allows robust conclusions to be drawn.

This has been clarified in the manuscript:

L566-567:
This process ensured the removal of non-bound or transiently bound biopolymers that could artificially elevate the quantity of bound biopolymer.

- Rheological tests: The authors indicate that the water content is adjusted to allow testing with the rheometer used, but they do not specify the criterion used for this adjustment. What is this criterion, and how can the rheological results be meaningfully compared across different formulations under these conditions?

We thank the reviewer for requesting clarification of the criterion used to adjust water content. The central objective of this study is the multiscale design of materials for extrusion-based 3D printing for construction. In this context, we consider it more meaningful to compare formulations at comparable processability rather than at identical clay-to-water ratios. For 3D-printing applications, controlling rheological behavior within a printable window is more critical than strictly fixing composition, as formulations with identical solid contents may exhibit vastly different flow and yield responses due to differences in earth mineral composition.

We have revised the manuscript to explicitly state this criterion and to clarify how rheological comparability across formulations is ensured from a processability-oriented perspective:

L605-607:
Quantitatively, we set a plastic viscosity limit of 10 Pa·s, corresponding to ~2200 Pa at 220 s^{-1}, near the rheometer limit; higher viscosities are also impractical for extrusion-based 3D printing.

- Engineered earth formulation: "Since chlorite clay, accounting for 9% of the natural earth composition (Extended Data Fig. 10f), was outside of the minerals explored for this study, it was replaced with 4.5% mica and 4.5% kaolinite…" How can one be confident that this substitution leads to a mechanical and rheological behavior comparable to that of the natural earth?

To ensure a well-defined engineered earth system that serves as a clear transitional step between controlled clay formulations and more complex natural earth materials, chlorite itself was not included directly, as it was not examined elsewhere in the study. Chlorite is a non-expanding 2:1:1 clay, consisting of a 2:1 (T-O-T) layer coupled to an interlayer brucite- or gibbsite-like hydroxide sheet, and therefore exhibits both permanent-charge silicate surfaces and hydroxyl-rich surface functionality. To preserve these functional characteristics within the engineered earth, chlorite was instead represented by a 50/50 mixture of mica and kaolinite: mica maintains the non-expanding 2:1 framework and permanent-charge behavior, while kaolinite provides hydroxyl-rich surface sites. Confidence that this substitution captures the relevant rheological behavior is provided by experimental evidence: a consistent alginate-induced dispersive response was observed across bulk rheological measurements, engineered earth formulations, heterogeneous natural earth at small scale printing, and heterogeneous natural earth printing at larger scale.

- Time-dependent behavior: Is a difference in hardening or setting rate observed between the different formulations? How do the rheological properties evolve with time and drying duration? Have the authors investigated the thixotropic behavior of the materials?

We thank the reviewer for these valuable and insightful comments regarding time-dependent behavior and thixotropy.

In this study, rheological measurements were selected with an emphasis on the immediate response after mixing (viscosity, yield stress, and storage modulus), which govern extrudability and buildability during extrusion-based 3D printing. The ability of the proposed multiscale framework to capture longer-term time-dependent effects, including structural build-up and thixotropy, will be the subject of future investigations.

L321-328:

Future studies can extend this approach to a broader range of processing parameters (i.e., structural build-up, thixotropy) and material properties (i.e., density, mechanical performance, thermal conductivity, and moisture resistance) under relevant environmental exposure conditions (i.e., humidity, temperature, and UV radiation), which are critical for the practical implementation of 3D-printed biopolymer-stabilized earth as a structural and/or insulation construction material. Importantly, the framework can be applied to optimize compressive strength, providing a pathway toward achieving the high strengths previously observed in bio-stabilized earth (up to 60 MPa) within 3D-printed structures.
